# Fluoride in the Central Nervous System and Its Potential Influence on the Development and Invasiveness of Brain Tumours—A Research Hypothesis

**DOI:** 10.3390/ijms24021558

**Published:** 2023-01-13

**Authors:** Wojciech Żwierełło, Agnieszka Maruszewska, Marta Skórka-Majewicz, Izabela Gutowska

**Affiliations:** 1 Department of Medical Chemistry, Pomeranian Medical University, Powstańców Wlkp. 71 St., 70-111 Szczecin, Poland; 2 Department of Physiology and Biochemistry, Institute of Biology, University of Szczecin, Felczaka 3c St., 71-412 Szczecin, Poland; 3 Molecular Biology and Biotechnology Centre, Institute of Biology, University of Szczecin, Wąska 13 St., 71-415 Szczecin, Poland

**Keywords:** fluoride, brain tumour, glioblastoma, invasiveness, multidrug resistance, environmental pollution

## Abstract

The purpose of this review is to attempt to outline the potential role of fluoride in the pathogenesis of brain tumours, including glioblastoma (GBM). In this paper, we show for the first time that fluoride can potentially affect the generally accepted signalling pathways implicated in the formation and clinical course of GBM. Fluorine compounds easily cross the blood–brain barrier. Enhanced oxidative stress, disruption of multiple cellular pathways, and microglial activation are just a few examples of recent reports on the role of fluoride in the central nervous system (CNS). We sought to present the key mechanisms underlying the development and invasiveness of GBM, as well as evidence on the current state of knowledge about the pleiotropic, direct, or indirect involvement of fluoride in the regulation of these mechanisms in various tissues, including neural and tumour tissue. The effects of fluoride on the human body are still a matter of controversy. However, given the growing incidence of brain tumours, especially in children, and numerous reports on the effects of fluoride on the CNS, it is worth taking a closer look at these mechanisms in the context of brain tumours, including gliomas.

## 1. Introduction

Macro- and microelements are one of the factors potentially implicated in the progression and malignancy level of different types of cancer [1,2,3,4,5,6,7,8,9,10,11]. This can be evidenced by the fact that the content of various mineral elements differs between cancerous and noncancerous tissue [12]. While the overall number of studies is small, it would appear that breast, thyroid, kidney, stomach, and colorectal cancers are the best studied in this regard [13,14]. Unfortunately, there are only a few papers focusing on brain tumours [12].

The few existing reports point to significant disturbances in mineral concentrations (including Cu, Zn, Mg, Br, Sr, Fe, Ca, P, S) in various brain tumours [15,16,17,18]. The observations usually reveal a significant increase or decrease compared with noncancerous tissue, but results are very difficult to compare and are often contradictory. There are even fewer reports from studies on heavy metals [19,20]. In the face of recent scientific evidence on the high risk and wide range of adverse effects of fluoride on the central nervous system, it may come as a surprise that none of the studies dedicated to mineral elements and brain tumours to date have investigated the effects of fluoride [21].

Fluorine is a trace element. The levels of trace elements in the brain are regulated in complex ways by brain barrier systems, such as the blood–brain barrier (BBB), blood–cerebrospinal fluid (CSF) barrier, choroidal blood–cerebrospinal fluid interface, and even the CSF–brain barrier [22]. Studies have shown that fluorine is able to cross the blood–brain barrier and enter the brain tissue. This disrupts the normal metabolic process of the brain, generates free radicals, and causes various toxic effects in the brain [21,23,24].

Primary CNS tumours account for nearly 12% of all cancers; GBM is the leading type of primary malignant CNS tumour, accounting for almost half of all primary malignant CNS tumours and approximately 57% of all diagnosed gliomas [25,26]. Many of the brain tumours described to date are characterised by an aggressive and invasive clinical course and a high degree of malignancy. Brain tumours are one of the most common types of childhood cancer [27]. Given the harmful effects of fluoride on the central nervous system (CNS) in children [24] and the rise in the incidence of childhood brain tumours since the mid-1980s [28], there seems to be good reason to take a closer look at the role of fluorine in the context of brain cancer. The latest controversies surrounding fluoride are related to its toxic effects on the developing brain [29]. The increasing incidence of gliomas of varying degrees of malignancy in the brain and brain stem among children and the potential role of unidentified environmental carcinogens support the need for further research [30,31].

## 2. Fluoride as an Environmental Toxin

Fluoride is regarded as an environmental pollutant associated with serious effects on the functioning of organisms and ecosystems [32]. Fluorine in its elemental form is practically not found on Earth, but it is present in the ecosphere in the form of fluorine compounds. They occur naturally in a wide variety of minerals in the Earth’s crust, from where fluorides are released into the soil and water through the Earth’s volcanic activity and rock erosion [33,34]. Fluoride may pose a threat to human health, which has been specifically documented for populations inhabiting industrialised areas. In these areas, soil and water fluoride levels are elevated due to release from anthropogenic sources. These include fertilizers, pesticides, and deposits of industrial air pollution. Sources of industrial fluoride emissions include combustion of fluoride-rich coal, petroleum refining, production of steel, clay, glass, enamels, bricks and ceramics, manufacture of chemicals, and nuclear fuels [32,33].

The fluorides distributed in soil, air, and water are accumulated by plants and animals [35]. Consequently, drinking water, which may also be artificially fluoridated as a public health measure, and food are major sources of fluoride uptake in humans. The degree of fluoride exposure is affected by the quality of food and water, the amount consumed, as well as individual variability [36,37,38,39].

The harmfulness of fluoride has been the topic of intense debate in the last twenty years. There are different opinions as to the role of fluorine as an essential element and the magnitude of its toxic effects on humans (especially through water fluoridation) [40], but a growing body of literature suggests that labelling fluorides as an environmental toxin appears to be correct.

The effects of fluoride on the human body can be considered in two ways. Low supply of fluoride interferes with dental enamel formation and promotes growth of cariogenic oral bacteria, leading to dental caries. Fluoride deficiency also causes bone demineralization [32,34,41]. On the other hand, through complex molecular mechanisms of fluoride action on the cellular level, acute and chronic exposure to elevated doses may trigger a broad spectrum of disorders, both physiological and developmental.

**Figure 1:**
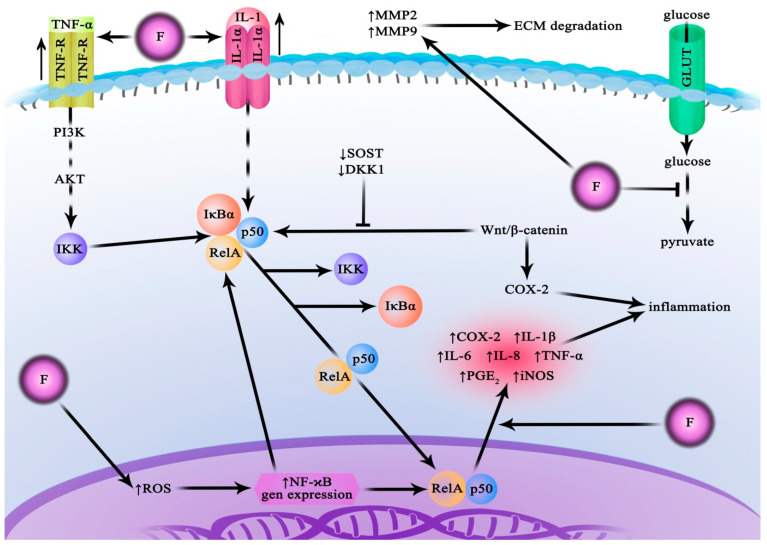
Fluoride action in normal cells. Fluoride affects normal cells in a pro-inflammatory manner, which is associated with the activation of the NF-kB pathway. As an inhibitor of glycolysis enzymes, it leads to disturbances in energy metabolism. AKT—protein kinase B; COX-2—cyclooxygenase-2; DKK1—Dickkopf-related protein 1; GLUT—glucose transporter; IKK—IκB kinase; Il—interleukin; iNOS—inducible nitric oxide synthases; IκBα—nuclear factor of kappa light polypeptide gene enhancer in B-cells inhibitor alpha; MMP—matrix metalloproteinase; NaF—sodium fluoride; NF-κB—nuclear factor kappa-light-chain-enhancer of activated B cells; p50—NF-kappa-B p105 subunit; PGE2—prostaglandin E2; PI3K—phosphoinositide 3-kinase; RelA—NF-kappa-B p65 subunit; ROS—reactive oxygen species; SOST—clerostin protein; TNF—tumour necrosis factor; TNFR—tumour necrosis factor receptor; Wnt/β-catenin—Wnt signalling pathway.

Fluoride has been shown to inhibit or activate numerous enzymes crucial for cell metabolism and signalling (Figure 1). It suppresses the activity of Mg-dependent enzymes, including those that catalyse glycolytic reactions. It has also been shown to inhibit pyrophosphatases, ATPases, acetylcholinesterase, and cytochrome c oxidase. On the other hand, stimulatory effects of fluoride have been observed in, for example, glycogen phosphorylase, aspartate transaminase, and tyrosine kinase [40]. Furthermore, fluoride influences intracellular signal transduction pathways by affecting signalling cascades involving, e.g., G proteins, adenylate cyclase, Hedgehog proteins, and transcription factors such as NF-κB and Nrf2 [42,43]. It has also been demonstrated to induce abnormal methylation in some regions of the genome [44]. Consequently, with increased exposure, fluorine compounds can exert toxic effects, including organelle damage, oxidative stress on the cellular level, cell cycle disruption, inflammatory cytokine secretion, induction of apoptosis, and disruption of synaptic neurotransmission [40,45,46]. Fluoride is also regarded as a potential endocrine disruptor leading to the development of thyroid dysfunction [47].

### Fluoride Neurotoxicity

**Figure 2:**
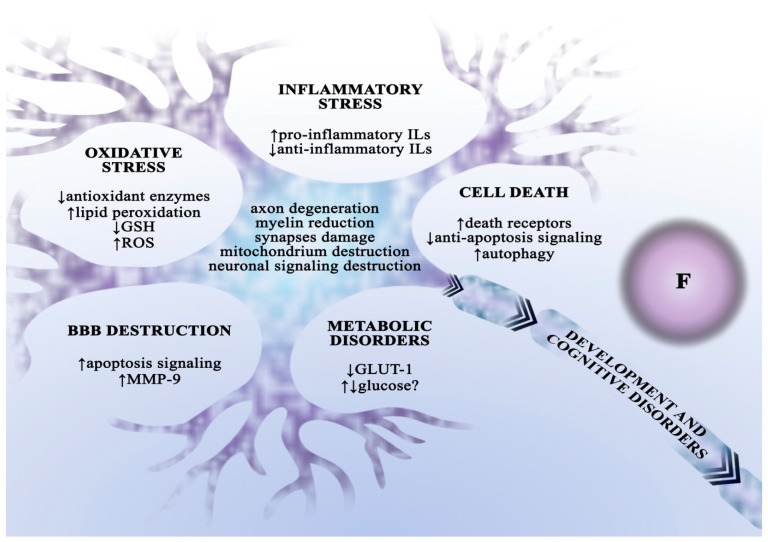
The neurodegenerative effect of fluoride. The toxic effect of fluoride on the central nervous system is multidimensional—it results from disturbances in metabolism regulation, synaptic functioning, the blood–brain barrier integrity, as well as oxidative stress and inflammation induction in neurons and microglia cells. These multidimensional interactions are believed to cause developmental and cognitive impairments in the body. ADHD—attention deficit hyperactivity disorder; BBB—blood–brain barrier; GLUT—glucose transporter; GSH—reduced glutathione; Ils—interleukins; ROS—reactive oxygen species.

One of the better-known toxic effects of chronic fluoride exposure is dental and bone fluorosis, manifested by structural abnormalities in dental enamel as well as bones, ligaments, and tendons [34]. Besides teeth and bones, fluoride accumulates in soft tissues, hence chronic exposure can cause damage to the liver, kidneys, cardiovascular system, and reproductive system [48,49,50,51,52,53,54]. Still, perhaps the most concerning data point to the significant role of fluoride as a neurotoxin, fluoride penetrates the BBB and alters the structure and function of nervous tissue [24,55,56,57]. Furthermore, in a study with a rat model, chronic fluoride exposure was shown to increase the levels of metalloproteinase 9 (MMP-9) and p53 protein, leading to cell apoptosis and damage of the blood–spinal cord barrier [58]. The neurodegenerative effects of fluoride are particularly critical in the early stages of biological development, which many authors attribute to its ability to cross the blood–placenta barrier [59]. Fluoride causes degenerative changes in all parts of the brain and in the spinal cord, including axon deterioration, myelin sheath degeneration, mitochondrial damage, and alterations in synaptic ultrastructure [56,60]. It also affects neurotransmitter metabolism and causes changes in the expression of neurotransmitter receptors [61,62,63]. Fluorine compounds impair energy metabolism of the brain, dependent primarily on the burning of glucose. Fluoride exposure may be associated with changes in the profile of proteins involved in energy metabolism [64], and researchers have suggested that impaired glucose metabolism in neurons is correlated with decreased expression of the GLUT-1 transporter [65]. On the other hand, increased glucose transport into brain cells has also been documented, although without changes in transporter expression, suggesting a compensatory mechanism in response to damage [66]. Chronic fluoride exposure also affects amino acid and lipid metabolism [61,67]. Neuronal damage as a result of exposure to high doses of fluoride is associated with the induction of cellular oxidative stress and inflammation. In vitro and in vivo studies have shown that fluoride increases ROS levels through lipid peroxidation, decreasing GSH levels, and suppressing antioxidant enzyme activity [68,69,70]. Fluoride exposure results in increased secretion of pro-inflammatory interleukins and decreased production of anti-inflammatory interleukins [42,68,71]. Fluoride-induced neuronal degeneration is associated with the activation of apoptotic signalling cascades [72], increased expression or higher levels of death receptors [73] and pro-apoptotic proteins [58,71,74], as well as caspase activation [68,73] and downregulation of anti-apoptotic protein expression [71]. Neuronal degeneration can also occur via autophagy (Figure 2) [75].

The described molecular-level changes leading to neuronal degeneration manifest themselves in developmental and cognitive disorders that have been observed both in animal models and in population studies. It has been observed that chronic fluoride exposure during the prenatal period and early life may manifest as deficits in learning and memory, reduced non-verbal intelligence (PIQ), and lower intelligence quotient (IQ) [24,76,77,78,79]. Some authors have also suggested that elevated fluoride levels are correlated with the risk of dementia [80] and ADHD prevalence [81]. It should be noted that many authors disagree with these conclusions, arguing that population-based studies are incomplete. They also note that many of the behavioural studies were conducted in animal models utilizing acute doses [21,82,83,84]. Nevertheless, as Till and Green [85] point out, the evidence is relatively new and should rather be regarded as a potential early warning.

## 3. Gliomas

Gliomas are primary brain tumours. Recent data indicate that these tumours are derived from neural stem cells (NSCs), NSC-derived astrocytes, and oligodendrocyte precursor cells (OPCs) [86]. In the CNS, there are three types of glial cells: astrocytes, oligodendrocytes, and microglia. Astrocytes are the most common type of glial cells in the CNS. They are star-shaped cells that are responsible for metabolic homeostasis and can acquire reactive phenotypes in response to pathogens or CNS injury. This process is very complex and its deregulation promotes cancer development [87].

### Description

Even though significant advances have been made in the last decade in the treatment of many types of cancer, the survival rate of patients with glioblastoma (GBM) is still around 14 months—in spite of effective diagnosis, advanced radiotherapy, targeted chemotherapy, and high-precision neurosurgical procedures [88]. The current standard of care for patients diagnosed with GBM is maximum safe surgical resection and combination radiotherapy. In addition, treatment involves oral administration of temozolomide (TMZ), a potent alkylating agent able to penetrate the BBB [89]. Treatment of GBM is difficult; tumour hypoxia is a common feature promoting multiple adverse mechanisms, including GBM cell resistance and invasiveness and infiltration of surrounding normal brain tissue. Moreover, the issue of drug delivery penetrating through the BBB is a challenge in the development of new drugs [90].

Metastasis in GBM is different from other types of aggressive cancers. While most cancers metastasize to other organs via the circulatory or lymphatic system, glioma cells rarely spread outside the brain and usually migrate extensively through the extracellular matrix, infiltrating normal brain tissue [91]. For this invasion to occur, glioma cells undergo a number of biological changes, acquiring motility and the ability to degrade the extracellular matrix (ECM), and transitioning into a mesenchymal phenotype [92]. Glioma cells change their shape and size and squeeze through the tight spaces of normal brain tissue. In addition, invasive glioma cells interact with many components of the extracellular matrix. Even though the ECM represents a physical barrier to glioma cell invasion, it also provides glioma cells with essential ligands to which glioma cells can bind and then use them to migrate [93]. In addition, the ECM can exert chemical effects on glioma cells. Several studies have shown that tumours affect surrounding stroma cells, causing reorganization of the structure and composition of the extracellular matrix. These changes in the extracellular matrix promote tumour growth and invasion [94]. In addition to their migratory ability, glioma cells must be able to pass through the physical ECM barrier. By degrading ECM proteins, an invasive pathway is formed. Many studies suggest the involvement of matrix metalloproteinases (MMPs), often overexpressed in glioma cells, in ECM degradation [95].

Moreover, the high radio- and chemoresistance of glioma cells is one of the main reasons for treatment failure. Numerous, often unconventional, defence mechanisms and the high heterogeneity of glioma cells within the tumour make GBM one of the world’s most lethal cancers [96,97,98].

## 4. Mechanisms of Drug Resistance in Glioblastoma

Temozolomide (TMZ) is the most widely used drug in the treatment of glioblastoma [90]. TMZ is an alkylating agent which induces DNA double strand breaks, resulting in cell cycle arrest leading to cell death. Due to its short half-life, TMZ is administered in large doses, and prolonged systemic administration leads to a range of adverse effects. Resistance to TMZ therapy is an important issue and also one of the main causes of treatment failure, suggesting that overcoming TMZ resistance is crucial to improve patient outcomes [99]. Although TMZ is a first-line chemotherapeutic, it yields a minimal increase in median overall survival, because of ‘innate’ resistance due to pre-existing factors or ‘acquired’ resistance, which develops during treatment. The leading mechanism of resistance in glioma cells is the high activity of O-6-methylguanine DNA methyltransferase (MGMT), which repairs TMZ-induced DNA damage and contributes to TMZ resistance [100]. TMZ resistance in GBM has also been linked to a number of cellular signalling pathways, including Hedgehog (HH) [101], NF-κB [102], Wnt/β-catenin (Wnt) [103], and Notch [104].

### 4.1. Hedgehog Signalling Pathway (HH/GLI1)

**Figure 3:**
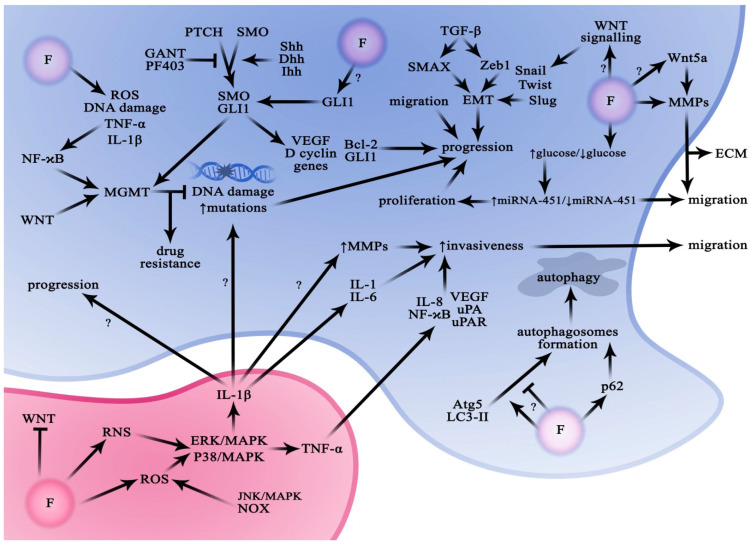
Effect of fluoride on signalling pathways in glioma cells. Although the data currently available is incomplete on many issues, it appears that fluoride may affect signalling pathways involved in the processes of apoptosis, autophagy, inflammation, and chemoresistance of glioblastoma cells. Thus, this compound has the potential to promote the growth and invasiveness of gliomas. Atg5—autophagy-related protein; Bcl-2—B-cell lymphoma 2 protein; Dhh—Desert hedgehog protein; ECM—extracellular matrix; EMT—epithelial-mesenchymal transition; ERK/MAPK—MAPK/ERK pathway; GANT—GLI antagonist; GLI1—zinc finger protein GLI1; Ihh—Indian hedgehog protein; Il—interleukin; JNK—c-Jun N-terminal kinase; LC3-II—microtubule-associated proteins 1A/1B light chain 3B; MGMT—O-6-alkylguanine DNA alkyltransferase; MMPs—matrix metalloproteinases; NaF—sodium fluoride; NF-κB—nuclear factor kappa-light-chain-enhancer of activated B cells; NOX—NADPH oxidase; p-62—ubiquitin-binding protein p62; PF403—Hedgehog signalling pathway repressor; PTCH—Protein patched homolog; RNS—reactive nitrogen species; ROS—reactive oxygen species; Shh—Sonic hedgehog protein; Slug—transcription factor Slug; SMAX—protein suppressor of Max2 1; SMO—Smoothened protein; Snail—zinc finger protein SNAI1; TGF-β—transforming growth factor β; TNF-α—tumour necrosis factor α; Twist—twist-related protein; uPA—urokinase-type plasminogen activator; uPAR—urokinase plasminogen activator surface receptor; VEFG—vascular endothelial growth factor; WNT—Wnt (Wingless/Int-1) proteins; Zeb1—zinc finger E-box binding homeobox 1.

The Hedgehog signalling pathway plays an important role in the development of the central nervous system and, in adult life, in maintaining normal neural tissue and the stem cell pool [105]. However, through its regulatory potential, it often plays a key role in tumourigenesis and progression of tumours, including gliomas [106]. Regulation of Hedgehog signalling activity is complex and occurs via specific ligands, including Sonic hedgehog (Shh), Indian hedgehog (Ihh), and Desert hedgehog (Dhh), which bind to the Patched receptor (PTCH) inhibiting a protein called Smoothened (SMO). The released SMO protects the glioma-associated oncogene homolog 1 (GLI1) protein from splicing. As a result, GLI1 can translocate into the nucleus and act as a transcription factor leading to increased expression of many genes, including GLI1, PTCH1, cyclin D, Bcl-2, and VEGF [106,107]. There are also alternative pathways of non-canonical GLI activation occurring independently of SMO, such as those activated by PI3K/AKT or MEK, [108,109]. The GLI protein family (GLI1, GLI12, and GLI3) is a group of transcription factors that contain zinc fingers. There is an additional truncated isoform of the GLI1 protein, known as tGLI1, that has been shown to stimulate the motility and invasiveness of glioma cells [110]. Additionally, several papers have linked the tGLI1 factor to enhanced tumour vascularity through upregulation of heparanase and VEGF expression (Figure 3) [110,111,112].

A study using the U251MG cell line showed that the HH/Gli1 signalling pathway regulates MGMT expression and chemoresistance to TMZ in human GBM, irrespective of the MGMT promoter methylation status [113]. A HH/Gli1 inhibitor (GANT61) was found to sensitize U87MG and U251MG glioma cells to TMZ treatment by enhancing the DNA damage effect, suppressing MGMT expression and the Notch1 pathway [114]. In TMZ-resistant glioblastoma with high expression of MGMT, the repression of the HH signalling pathway by PF403 also reduced MGMT expression [115].

#### The Role of Fluoride in the Regulation of the Hedgehog Signalling Pathway

There is currently no direct evidence that fluorine compounds can lead to the activation of the HH-GLI1 pathway in GBM cells. Nevertheless, there are several reports which indirectly suggest that this influence cannot be completely ruled out. In a study by Wang et al., disrupted osteoblast function and impaired bone formation were demonstrated after excessive fluoride exposure (MC3T3-E1 cell line, 8 mg/L NaF for 7 days). The observations included a marked increase in HH and Notch pathway activity, as well as increased levels of insulin, TGF-β, and VEGF [116]. Another study in Wistar rats exposed to NaF in drinking water (50 mg/L for 6 months) showed a significant increase in mRNA and protein expression of Shh, SMO, and GLI1 in hepatocytes [117]. Activation of the HH signalling pathway and excessive expression of downstream target genes may be responsible for chondrocyte damage in chronic fluorosis in rats [118]. A recent study (2021) on rat osteoblasts clearly shows a significant dependence of Ihh, SMO, and GLI2 expression in osteoblasts on the applied dose of fluoride (Figure 3) [119].

### 4.2. Nuclear Transcription Factor κB (NF-ĸB) Pathway

NF-κB signalling pathways can be activated by a range of diverse factors, such as growth factors, ROS, oncogenic stress, DNA damage, ionizing radiation, UV, various cytokines (TNF-α and IL-1β), and many others [120,121,122,123,124]. Nuclear transcription factor κB (NF-ĸB) controls the expression of numerous genes associated with tumour invasiveness and involved in proliferation, apoptosis, angiogenesis, and metastasis. Abnormal NF-κB activity plays an important role in promoting tumour invasion and response to therapy [125]. Neovascularization in GBM is critical for supporting the growing tumour, and stimulation of this process is dependent on several NF-κB target genes, including VEGF, IL-6, and IL-8 [126,127]. NF-κB also plays an important role in regulating tumour cell infiltration by controlling the expression of many adhesion molecules, such as fibronectin and vitronectin [128] responsible for the invasion of matrix metalloproteinases such as MMP-2 and MMP-9 [129,130]. In addition, NF-κB activation may promote epithelial-mesenchymal transition (EMT), important for tumour invasion and treatment resistance [121,131,132,133,134].

Excessively activated NF-κB features in the major inflammatory transcription pathway associated with TMZ resistance in GBM [135]. However, the relationship between the NF-κB pathway and MGMT expression in GBM cells is unclear. Given that both MGMT and NF-κB are strongly expressed in the TR/U251 glioma cell line, a link between them and TMZ resistance is likely. The IκBα inhibitor, BAY 11-7082, in combination with TMZ, significantly suppressed MGMT levels in TR/U251 cells and promoted the initiation of TMZ-induced apoptosis, suggesting that NF-κB plays a key role in the regulation of MGMT expression [136]. It is also hypothesised that TMZ-induced DNA damage activates ataxia telangiectasia mutated (ATM) kinase, which simultaneously triggers MGMT repair and inappropriate activation of NF-κB [137].

#### The Role of Fluoride in the Regulation of the NF-κB Pathway

Several in vitro and in vivo studies have shown that fluoride may play an indirect role in the regulation of the NF-κB pathway. Increased NF-κB expression induced by fluoride has been observed, among others, in monocytes [138], macrophages [139], peripheral blood mononuclear cells [140], and the human lung epithelial cell line (1.0–3.75 mM) [43]. The results of a study in mice exposed to fluorine compounds showed that NaF at concentrations exceeding 12 mg/kg induced renal inflammatory responses through the activation of NF-κB, decreasing the expression of anti-inflammatory cytokines (IL-4 and IL-10), and increasing the levels of PGE2, iNOS, COX-2, IL-6, and IL-8 compared with controls [141]. Another study in mice demonstrated that inflammation also develops in the liver, in association with the activation of the MAPK and NF-κB pathway, and with increases in IL-1β, IL-6, IL-8, COX-2, and MCP-1 [42]. Similar observations have been reported in the spleen [142]. It has further been shown that fluoride can activate the NF-κB pathway by promoting TNF-α synthesis [143] and inhibiting the expression of vitamin D receptor (VDR) [144], involved in downregulating NF-κB activation [145]. There are also several papers describing the mechanism of action of NaF on various brain tissues [146,147,148,149], which nevertheless remains unclear (Figure 3).

### 4.3. Wingless/Int1 Trail (Wnt) Pathway

The Wingless/Int1 (Wnt) signalling pathway plays an important role in the development of the central nervous system. Wnt signals through two separate pathways, canonical (β-catenin dependent) and non-canonical (β-catenin independent). The Wnt system is often overactive in GBM tumours, enabling the proliferation and invasiveness of tumour cells [150]. The canonical Wnt pathway promotes GBM invasion by maintaining cancer stem cells and promoting EMT processes [151]. Activation of the Wnt/β-catenin pathway results in increased expression of EMT-promoting transcription factors such as Twist, Snail, Slug, and Zeb1 [152]. The canonical pathway has also been linked to the development of resistance to chemotherapy and radiotherapy [153]. Non-canonical Wnt activation, on the other hand, is an important regulator of cell motility and tissue polarity, which controls the migration of neuronal and epithelial cells [154], as well as GBM [155]. Research has shown that Wnt5a, a non-canonical Wnt ligand, appears to be a critical master regulator of the invasive capacity of human glioma stem cells (GSCs) in vivo [156]. Wnt5a enhances glioma cell migration by regulating the expression of MMP-2, which is involved in ECM degradation [157].

The Wnt/β-catenin pathway regulates MGMT gene expression [158] and its inhibition may be a promising molecular target for GBM therapy. Some inhibitors of the Wnt/β-catenin pathway, such as salinomycin, celecoxib, and Wnt-C59, restore TMZ sensitivity in resistant GBM cells by reducing MGMT expression in GSCs.

#### The Role of Fluoride in the Regulation of the Wingless/Int1 (Wnt) Pathway

The effect of fluoride on the activation of Wnt signalling in cancer cells is unknown. However, there are several studies implicating fluoride in Wnt signalling in healthy tissues (Figure 3). Fluoride was shown to increase the production of IL-6, TNF-α, and ROS, promoting inflammation and oxidative stress with concomitant inhibition of the canonical Wnt signalling pathway activity and stimulation of the NF-κB pathway activity in BV2 microglial cells [159]. Other observations included elevated levels of a Wnt antagonist, Dickkopf Wnt signalling pathway inhibitor 1 (DKK1), NF-κB activation, and increased production of pro-inflammatory mediators IL-6, TNF-α, and ROS. In addition, the results of another study indicate that long-term exposure to elevated fluoride levels can decrease the concentrations of sclerostin (SOST) and DKK1, physiological Wnt/β-catenin pathway inhibitors [72]. This association is also supported by research showing that increasing exposure to fluorine and arsenic was accompanied by a gradual increase in the activation of the Wnt/β-catenin pathway, while DKK-1 content significantly decreased [160].

A study by Luo et al. demonstrated for the first time that activation of the Wnt9a/b-catenin/CyclinD1 pathway in osteoblasts is induced by fluoride exposure [161]. Another study showed that NaF activated both canonical and non-canonical Wnt signalling pathways in an ameloblast cell line in vitro. Gsk-3β and Axin1 decreased significantly upon stimulation with 1.5 mM NaF, whereas Dvl3 was significantly increased. The levels of Wnt3a and Wnt5a, the canonical and non-canonical Wnt family proteins, significantly increased in response to NaF treatment [162]. It has also been confirmed that both Wnt and Rho pathways were upregulated by 1.5 mM NaF [163]. In addition, excessive fluoride intake (5–50 ppm F^−^) in rats led to the stimulation of calpain-1 (a proteolytic enzyme), which was accompanied by a significant decrease in RhoA levels in the cytoplasm of hippocampal cells and a high increase in its expression in cell membranes [164]. 

### 4.4. Notch Signalling Pathway

Notch (Notch-1, 2, 3, and 4) with ligands (Jagged-1, Jagged-2, and Delta-like-1, 3, and 4) regulates core cellular processes, including proliferation, apoptosis, migration, self-renewal, and differentiation of many cell types, and therefore plays a fundamental role in CNS development [165]. Over the years, deregulated Notch signalling has also been detected in several solid tumours, including brain tumours [166].

Both Notch mRNA and protein expression is higher in GBM cells than in normal brain cells [167], and Notch was found to be more expressed in peritumour-tissue GSCs compared with tumour-core GSCs [168]. Notch can stimulate β-catenin and NF-κB signalling through PI3K/AKT activation in glioma cells. This often correlates with increased expression of VEGF, Snail, Zeb1, and vimentin, and downregulation of the tumour suppressor gene PTEN, which consequently promotes cell invasion and migration [149,169]. Activation of this pathway is often associated with radioresistance of GBM [170]. It is observed in patients with shorter survival times and seen as a negative prognostic factor [171,172].

#### The Role of Fluoride in the Regulation of the Notch Pathway

There are no reports on the role of fluoride in regulating the Notch pathway activity in humans. Few animal experiments have shown that excessive exposure to fluoride (50 and 100 mg/L NaF) decreases mRNA and protein expression of Notch-3 and Jagged-1, as well as the expression of the target gene Hes-5 in rats, suggesting that fluoride may inhibit the Notch signalling pathway [173]. However, the topic should be considered unexplored and in need of further clarification.

## 5. Autophagy—Its Role in the Pathogenesis of Cancer

Autophagy is a highly conserved cellular process found in all eukaryotes which plays an important role in maintaining cellular homeostasis. Its functions include the degradation of damaged or unwanted intracellular proteins or cellular organelles. Furthermore, under stress conditions associated with, for example, nutrient deprivation or hypoxia, autophagy leads to the degradation of cellular components to provide amino acids or energy-rich biomolecules [174]. There is evidence that autophagy may also be involved in preventing oxidative stress, DNA damage, and oncogenic cell transformation [175]. However, it is believed that depending on the context, autophagy may inhibit carcinogenesis or, conversely, promote cancer development by affecting different aspects of tumour cell growth. There is ample evidence that autophagy can regulate pro-growth signalling and metabolic transformation of cancer cells, promoting tumour growth, and also contribute to developing resistance to chemo- or radiotherapy [175,176]. In light of recent studies, the autophagy process has emerged as a very interesting molecular target for the development of novel anticancer therapies in many types of cancer [177].

Unfortunately, the role of autophagy in gliomas is still not fully understood and remains a matter of ongoing debate. Based on studies using other solid tumour models and the few findings on glioma, autophagy may play a role, in a context-dependent manner, in tumour initiation, development and response to treatment, or in the inhibition of various aspects of tumour progression. By regulating receptor tyrosine kinase signalling and trafficking, as well as providing metabolites to fuel unconstrained proliferation, autophagy can accelerate tumour growth [178]. There is evidence that autophagy can regulate the epithelial–mesenchymal transition (EMT) of glioma cells and also influence oncogenic Met signalling [179]. By degrading proteins of the major histocompatibility complex (MCH-I), autophagy may assist in cancer cell evasion of the immune system [180].

### The Role of Fluoride in Autophagy Regulation

There is growing evidence that fluoride may play an important role in initiating autophagy processes through different types of signalling in various cell lines and systems in vivo (Figure 3). Because of the role played by fluorine in the body, the largest body of data comes from studies on bone and dental tissues. In a study using osteoblast-like cells, exposure to NaF was shown to enhance autophagy by upregulating the gene expression of sirtuin 1 (SIRT1). The increase in SIRT1 promotes deacetylation of another protein, FoxO1, and triggers a downstream cascade of reactions that suppress NaF-induced apoptosis and enhance autophagy [181]. Another study in the MC3T3-E1 osteoblastic cell line suggests that NaF may induce endoplasmic reticulum (ER) stress, leading to the initiation of both autophagy and apoptosis [182]. Recent reports from studies on osteoblasts isolated from Sprague-Dawley rats and treated with NaF solutions confirmed increased expression of autophagy-related genes (LC3A and Beclin1) compared with controls [183]. Another experiment with ducks exposed to long-term contact with fluoride showed that an excess of this ion triggered autophagy (elevated markers: Beclin 1, mTOR, Pakin, Pink) and caused cartilage damage in the tibia [184]. In turn, a study by Ma et al. [185] showed that NaF significantly downregulated the expressions of mTOR signalling pathway-related genes, including PI3K, AKT, mTOR, 4EBP1, and S6K1, in the mouse ATDC5 chondrogenic cell line. The mRNA and protein levels of autophagy-related genes, LC3, Beclin1, and p62, were significantly changed after NaF treatment, promoting autophagy in ATDC5 cells. The findings presented by Suzuki and Bartlett [186] show that fluoride initiates autophagy to protect ameloblast cells (LS8) from exposure to the mineral by increasing SIRT1 expression, inducing SIRT1 phosphorylation, and increasing the expression of autophagy-related genes (Atg5, Atg7, and Atg8/LC3). The experiment repeated in rats confirmed the results obtained in vitro [186]. In another study in a rat ameloblast line, using both in vitro and in vivo models, an increase in the amount of autophagosomes was observed, as well as increased LC3 and Beclin1 expression, proportional to the dose of fluoride administered [187].

Deregulation of autophagy associated with fluoride has also been demonstrated outside the teeth and skeletal system. In rat kidney epithelial cells (NRK-52E) treated with high concentrations of NaF, in the first 12 h of exposure only, autophagy was induced, and after 24 h, the markers associated with apoptosis or necrosis were increased too [188]. In a study conducted on the offspring of rats whose mothers were administered fluorine and/or arsenic, a significant increase was observed in renal tissue in the expression of a number of genes closely related to autophagy, i.e., LC3, LC3I, LC3II, Beclin-1, ULK1, Atg13, and Atg14, with a concomitant decrease in mTOR and Bcl-2 levels [189]. A similar study undertaken by another team confirmed increases in the number of autophagosomes and in the expression of autophagy markers in kidney tissue of the offspring of dams (mother rat) exposed to fluorine compounds [190]. Fluoride also increased the levels of mRNA and protein expression of autophagy markers LC3, Beclin1, and Atg 5 in primary Leydig cells [129]. A similar effect was observed by Liu et al. when they examined the testes of rat offspring exposed to the mineral. They demonstrated that fluoride can modulate autophagy, causing increased levels of Beclin 1 and LC3 and decreased p62 expression [52]. An increase in autophagy markers (LC3, Beclin1, Atg16L1, Atg12, Atg5) was also observed in mouse splenocytes exposed for 42 days to NaF administered in water [191]. There is also evidence that fluoride induces apoptosis and autophagy via the IL-17 pathway in rat liver [192].

There is little research on the effects of fluoride on autophagic processes within the brain. What is more, the results of individual studies are inconsistent. Human neuroblastoma cells (SH-SY5Y) treated with different concentrations of fluoride exhibited abnormalities related to autophagy. The amounts of autophagic vesicles were markedly decreased in cells treated with higher concentrations of NaF (40–60 mg/L) compared with controls. In addition, the expression levels of autophagy-relevant proteins (Atg5 and LC3-II) were markedly lower, while p62 protein was significantly increased [193]. Further in vivo studies suggested that at lower NaF concentrations (<30 mg/L), the mineral can promote autophagy through a compensatory mechanism (increased expression of Beclin1, LC3-II and p62 in the hippocampus) [60], while higher NaF concentrations (>40 mg/L) can inhibit autophagy [75]. On the other hand, immunohistochemical analysis of the brain tissue of rats treated with NaF in drinking water showed that NaF administration at 25–100 mg/L induced autophagy, with a strong increase in Beclin1 protein in the hippocampal regions *gyrus dentatus* (DG) and *cornu ammonis* (CA1) [194].

A growing body of research now confirms that fluoride may play an important role in modulating autophagy-related processes. Nevertheless, it should be noted that there are few studies investigating the effects of fluoride on autophagy within the brain. However, given the potentially crucial role of autophagy in the progression and invasiveness of gliomas, future studies aimed at clarifying the role of fluoride in this process seem to be well-justified.

## 6. Glioma Microenvironment

The tumour microenvironment plays an important role in glioma progression. Microglia are the resident immune cells of the brain and are easily activated by a variety of foreign substances, including environmental toxins. It has been shown that tumour-associated microglia may be responsible for promoting glioblastoma invasion [195]. Within glioma, the functions of both microglia and macrophages are altered and they can enhance tumour-mediated immunosuppression as well as promote tumour invasiveness [196]. Expression of MMPs, which degrade the extracellular matrix in the glioma microenvironment, is associated with increased GBM cell invasion and enhanced angiogenesis. In particular, the activation of the CX3CL1/CX3CR1 system (fractalkine and its receptor) has been shown to upregulate the expression of metalloproteinases, both those secreted outside the cell (MMP-2, MMP-9) and membrane-bound (MT1-MMP, MMP14) [195,196,197]. It has also been suggested that microglia may promote angiogenesis by regulating VEGF [198]. In addition, other factors secreted by microglia residing within the GBM, such as the epidermal growth factor (EGF), IL-1β, IL-6, and IL-8, can activate receptors on GBM cells, promoting tumour invasion [199].

### The Role of Fluoride in Modulating the Glioma Microenvironment

There is evidence that fluorine compounds can activate microglia, leading to the release of numerous pro-inflammatory cytokines. In studies on the BV-2 microglial cell line, fluorine was shown to enhance oxidative stress by inducing the formation of reactive oxygen species (ROS) and reactive nitrogen species (RNS) [70], consequently leading to the release of pro-inflammatory cytokines such as IL-1β and TNF-α [200]. In vivo studies confirm these findings. In rats exposed to NaF in drinking water (60–120 ppm of F^−^), microglia activation promoted the secretion of the cytokines IL-1β and TNF-α via ERK/MAPK and P38/MAPK signalling pathways. Furthermore, fluorine-induced ROS production was involved in the activation of the JNK/MAPK pathway and NOX [201]. Fluoride also causes excessive activation of microglia in mouse hippocampus [202].

Cytokines IL-1β and TNF-α are generally recognized as inhibitors of glioma growth and associated with better prognosis [203,204]. However, some more recent studies cast a different light on these cytokines. It is possible that DNA damage induced by IL-1β stimulates relative protection of CSCs with concomitant accumulation of potential oncogenic mutations [205]. Additionally, a study by Sarkar and Yong showed that increases in IL-1β and TNF-α levels were positively correlated with glioma cell invasiveness and a corresponding elevation of MMP-2 and MMP-9 proteins [206]. A study using the U251MG human glioma cell line showed that IL-1β stimulates the production of IL-6 and IL-8, which in turn promotes cell invasion [207]. Most glioma cells are insensitive to the proapoptotic effects of TNF-α [208]. Nevertheless, TNF-α was observed to promote glioma cell motility and invasion by activating NF-κB [209], by increasing mRNA expression of uPA and uPAR genes in the U373MG cell line [210], and by modulating VEGF and IL-8 gene expression in the U251MG cell line [211]. These results indicate that the impact of IL-1β and TNF-α on glioma progression is not clear and may involve different intracellular pathways. The potential role of fluoride in this context seems interesting (Figure 3).

## 7. Involvement of Metalloproteinases in Glioma Development

Experimental and clinical studies confirm that elevated levels of matrix metalloproteinases (MMPs) are implicated in brain tumour progression. Elevated MMP levels, including MMP-1, -2, -7, -9, -11, -12, -14, -15, -19, -24, and -25 have been observed in malignant glioma samples from patients, suggesting that malignant progression is correlated with MMP expression [212]. MMPs have been shown to play a key role in the mechanisms of glioma invasion [130]. Among other things, MMPs participate in remodelling and degradation of the ECM (collagen, fibrinogen, proteoglycans) [213]. Moreover, MMPs are involved in angiogenesis, tumour infiltration, and further metastasis. They may also affect the metabolism of various cytokines, chemokines, and growth factors [214,215,216,217]. In the case of GBM, the MMPs of greatest interest are MMP-2 and -9, due to the close association with tumour growth and malignant progression [212]. Additionally, the expression of MMP-2 and MMP-9 was observed to be significantly higher in recurrent gliomas than in primary gliomas, and correlated with increased resistance to radiotherapy [217].

### The Role of Fluoride in the Regulation of Metalloproteinase Activity

Several reports indicate that fluoride, depending on concentration, can interfere with MMP levels in various tissues (Figure 3). Low doses slightly increase MMP-2 and -9 activities in preosteoblasts representing the MC3T3-E1 murine cell line after 24 h [218]. In situ administration of NaF (150 mg/L) to rats resulted in a significant increase in protein and mRNA expression levels of MMP-9 in uterine tissue [219], and an increase in MMP-9 and IL-17 in the cardiac muscle [220]. Chronic fluoride exposure upregulates the expression of MMP-9 and induces BBB damage and neurocyte changes [58]. It also disrupts the balance in gene and protein expression of MMP-2 and MMP-9 proteins and their inhibitors (TIMP2 and TIMP3) in brain structures such as the cerebellum, striatum, prefrontal cortex, and hippocampus [146].

## 8. Glial Defence Mechanisms against Metabolic Stress (Glucose)

The capacity to change phenotype and, consequently, to regulate migration, proliferation, survival, and angiogenesis are key mechanisms enabling neoplasms to resist adverse conditions such as metabolic stress. Depending on the prevailing conditions, glioma cells may adopt one of two phenotypes: higher proliferative activity with enhanced angiogenesis, or higher migratory activity with attenuated proliferative ability [221]. There are several theories on the modulation of glioma cell behaviour in response to hypoxia and glucose deprivation. One of them is the regulation of carboxypeptidase E, a neuropeptide-processing enzyme with anti-migratory and pro-proliferative effects [222]. Other research showed that miRNA-451 controls the balance of cell proliferation and migration in different glioma cell lines in response to glucose fluctuations [223,224]. When glucose is abundant, miRNA-451 is expressed in glioma cells, promoting proliferation, whereas low glucose levels are associated with miRNA-451 downregulation, resulting in a phenotype with increased glioma cell migration. In addition, high glucose levels may promote GBM progression by enhancing the function of chemoattractant and growth factor receptors (Figure 3) [225].

### The Role of Fluoride in the Regulation of Metabolic Stress (Glucose)

This is an issue of interest in the context of the effects of fluorine compounds on glucose metabolism. Studies in both humans and animals have shown that excessive fluoride intake alters blood glucose levels by affecting the regulation of metabolic pathways and release of hormones involved in carbohydrate metabolism [226]. A population-based study on the consumption of fluoridated tap water showed that additional fluoridation of tap water (0.7–1.2 ppm) was associated with an increase in the incidence and prevalence of diabetes from 2005 to 2010 in the United States [227]. In a study evaluating the effects of low-level fluoride exposure in drinking water (NaF 10 mg/L) in female NOD mice, there was a marked increase in fluoride levels and a 20% reduction in plasma glucose levels compared with controls [228]. This is correlated with earlier findings, showing a reduction in serum glucose levels in the offspring of mother rats given NaF orally (40 mg/kg) [229]. Dissimilarly, rats given NaF in drinking water (15 mg/L and 100 mg/L) presented a significant increase in plasma glucose and insulin resistance [230], as well as an increase in serum insulin coupled with a decrease in serum glucagon [231]. Potential changes in glucose uptake in the brain are an additional consideration. Studies in rats have shown that NaF decreases the expression of GLUT1, a glucose transporter in the brain, but the results of the few existing studies are conflicting. Some point to decreased expression of the GLUT1 glucose transporter and reduced glucose uptake into the brain [65], while others report compensatory increases in glucose uptake in the brain and peripheral tissues without significant changes in GLUT1 and GLUT3 expression [66]. Due to the paucity of data on the molecular mechanism underlying the effects of fluoride on brain glucose uptake, transport, and metabolism, it is difficult to clearly assess its role in glioma progression and invasiveness. However, given the significance of glucose levels in the phenotype adopted by glioma cells and the role of fluoride in glucose regulation, an indirect effect of this mineral seems plausible.

## 9. Involvement of Insulin and Insulin-like Growth Factor (IGF-1) in Glioma Development 

Insulin and insulin-like growth factor (IGF-1) signalling pathways are complex systems involving key regulators of cell transformation, growth, and cell cycle progression. Hence, their deregulation is often implicated in the development of many cancers, including brain tumours [232]. InsR and IGF1R receptors are commonly expressed in GBM tumours. Stimulation of these receptors promotes glioma cell proliferation and migration through negative or positive modulation of PI3K/AKT/mTOR and RAS/RAF/MEK/ERK signalling pathways [233,234]. Based on this, it is suggested that the interaction between ligands and InsR and IGF-IR triggers the progression of low-grade glioma to GBM [235]. Targeting both InsR and IGF1R with dual inhibitors has shown good results and appears to be one of the promising new treatment strategies [232,235].

### The Role of Fluoride in the Regulation of Insulin and Insulin-like Growth Factor

According to some studies, fluorine compounds may affect insulin metabolism. Low levels of fluoride exposure enhance insulin sensitivity [236]. In rats, plasma insulin levels increased in proportion to the fluoride concentration in drinking water [231,237]. Furthermore, fluoride stimulated the mRNA expression of InsR in the MC3T3-E1 osteoblastic cell line [231]. Finally, patients with endemic fluorosis were shown to exhibit significantly higher fasting insulin levels, and this effect could be reversed once the level of fluoride in drinking water was reduced [238].

It has been shown that in some cases, fluoride can activate the insulin-like growth factor (IGF-1) pathway and significantly increase serum IGF-1 levels [239]. Although the molecular mechanism of this process is not understood, it is known that fluoride at very low concentrations (1–10 μM NaF) stimulates PGE2 synthesis [240]. An increase in PGE2 can stimulate IGF-1 synthesis through a cyclic AMP/PKA pathway [241].

## 10. The Role of Transforming Growth Factor β in Glioma Metabolism

Increased transforming growth factor β (TGF-β) signalling activity is associated with glioma invasion due to its effect on cell migration. TGF-β induces EMT by activating SMAD2 and ZEB1, leading to enhanced motility and invasion (Figure 3) [92]. TGF-β is also a key regulator of glioma stem cells (GSCs). Recent studies have highlighted that TGF-β plays an essential role in the upregulation of the transcription factor Sox9, which promotes migration and invasion of glioma cells [242]. Furthermore, high TGF-β2 expression is associated with poor clinical outcomes in GBM patients [243].

### Effects of Fluoride on TGF-β

There is some evidence that TGF-β plays an important role in the body’s response to fluoride toxicity. In vivo and in vitro experimental studies on fluorosis have shown that fluoride upregulates the mRNA and protein expression of TGF-β1 in bone cells [244,245]. TGF-β1 is also known to play a mediating role in NaF-induced autophagy in mouse osteoblast cells [246]. In studies on human osteoblasts, NaF was observed to activate the TGF-β1/Smad2/3/CyclinD1 axis [247]. Animal studies demonstrated an increase in TGF-β levels in periodontal soft tissues [248] and an increase in TGF-β expression through an increase in IL-17A expression in testes [249]. Importantly, calcitonin, a hormone secreted by the parafollicular cells of the thyroid, was found to be a potent stimulator of TGF-β1 mRNA and protein expression [250].

Epidemiological studies have shown calcitonin to be strongly induced in humans upon exposure to fluorine compounds [251,252]. Dissimilarly, TGF-β1 expression in rat ameloblasts in response to NaF was significantly lower than in the control group [253]. These results support the findings of Suzuki et al., who showed that fluoride significantly decreased TGF-β1 transcript levels in rat tooth enamel [254]. Fluoride may also impair signal transduction between the epithelia and mesenchyma by inhibiting TGF-β3 expression in ameloblasts [255]. Analysis of microRNA expression profiling in fluoride-exposed MC3T3-E1 cells revealed that fluoride treatment affects numerous pathways, notably including TGF-β, Wnt, Hedgehog, and VEGF [116].

## 11. The Role of Thyroid Hormones in Glioma Development

Thyroid hormones and the individual steps of their genomic and non-genomic modes of action are disrupted in GBM. There is a well-established hypothesis that these disruptions have an effect on important pathways involved in the regulation of growth, proliferation, differentiation, and apoptosis of GBM cells, including the EGFR/PTEN/Akt/mTOR pathway, the TP53/MDM2/pl4ARF pathway, the P16/RB1 pathway, and the IDH/HIF-1 pathway. This has been well-described in a comprehensive review by Nauman [256].

Furthermore, thyroid hormones may exhibit proangiogenic properties by stimulating VEGF expression [257]. They can also activate microglia, stimulating microglial migration, motility, and phagocytosis [258]. Thyroid hormones have also been shown to induce MMP-9 gene expression in ovarian cancer and myeloma cells, promoting metastasis [259]. One of the factors responsible for chemoresistance in GBM is membrane protein P-glycoprotein (MDR1). Thyroid hormones are known to stimulate the transcription of the gene for this protein and affect its activity through the integrin receptor [260].

Pharmacologically induced hypothyroidism has been observed to achieve long-lasting regression of GBM, significantly prolonging patient survival, which indisputably confirms the involvement of thyroid hormones in the development and treatment of GBM [261]. However, there is still very limited knowledge, especially in the clinical area, in this matter [262,263].

### Effects of Fluoride on the Production of Thyroid Hormones

Epidemiological studies carried out in different geographical regions have demonstrated that fluoride exposure increases serum thyrotropin (TSH) levels in humans [264,265,266]. TSH stimulates thyroid follicular cells to produce thyroglobulin (Tg), triiodothyronine (T3), and thyroxine (T4) [267]. As discussed in a comprehensive review [256], the activity of thyroid hormones affecting various pathways involved in glioma progression and invasion (including p53, HIF-1α, PI3K, EGFR, VEGF), the therapeutic efficacy of chemical hypothyroidism, and the effect of fluoride on thyroid hormone levels appear to be worthy topics for investigation.

## 12. The Role of Glutamate in Gliomas

Migrating glioma cells undergo changes in shape and volume in order to facilitate their movement through the very narrow and tortuous extracellular spaces of the brain [268]. High extracellular glutamate levels serve as an essential autocrine/paracrine signal in tumour invasion through binding and activation of Ca^2+^ permeable AMPA receptors (AMPA-R). Most healthy neuronal cells contain a GluR2 subunit which prevents Ca^2+^ from passing through the AMPA-R channel pore. Gliomas, on the other hand, mainly express the GluR1 subunit in combination with GluR3 or GluR4 [269]. Overexpression of GluR1 results in an increase in glioma adhesion to ECM components such as collagen. Furthermore, AMPA-R proteins accumulate at focal adhesion sites, where they may mediate interactions between the ECM and integrins [270]. Ca^2+^ influx is essential in promoting cell motility and invasion, while the absence of an additional GluR2 subunit critically influences Ca^2+^ permeability [269] and is associated with a poor prognosis [271].

One of the treatment strategies being investigated is the stimulation of GluR2 expression in glioma cells. Experiments in C6 glioma cells and in a rat model showed that propofol significantly inhibited the viability, invasiveness, and migration of glioma cells by increasing the expression of GluR2 [272,273]. It has also been demonstrated that GluR2 inhibits proliferation by inactivating Src-MAPK signalling and induces apoptosis through caspase 3/6-dependent activation in glioma cells [274]. Perampanel treatment of human glioma cell lines U87 and U138 resulted in an increased GluR2/3 subunit expression and promoted apoptosis [275].

### Effects of Fluoride on Glutamate Metabolism

One of the few papers addressing this subject showed that maternal exposure to NaF (25, 50, and 100 mg/L) during gestation and lactation significantly reduced mRNA expression of the glutamate receptor GluR2 in the hippocampus of mouse pups. Otherwise, no significant changes in GluR1 and mGluR5 mRNA expression levels were observed [63]. Similar results were obtained in another study, which found that fluorine compounds activated microglia, stimulated the secretion of inflammatory factors, and strongly decreased GluR2 levels in the rat hippocampus [201]. If fluorine compounds can reduce GluR2 levels in glioma cells, they may indirectly promote their invasive potential.

## 13. Conclusions and Perspectives

Fluorine is an environmental pollutant, which upon entering the human body disrupts many of its processes. Its impact on many organs, including bones, liver, pancreas, lungs, heart, skeletal muscles, and kidneys, can no longer be denied. Furthermore, the ability of fluoride to cross the BBB means that it may also interfere with metabolic processes in the central nervous system, which has been supported by the few studies investigating the role of fluoride in the brain. However, there are virtually no well-documented studies demonstrating a direct effect of fluoride on the development, invasiveness, or resistance of brain tumours, including gliomas. The scant reports from in vitro studies in neuronal cell lines and in vivo studies in rodents, as well as findings referring to other tissues and organs, including human models, allow for the formulation of some tentative questions and hypotheses on the adverse effects of fluoride in the context of brain tumours. What is more, these findings suggest that the role of fluoride in this process may be indirect rather than direct, including the effects exerted on normal cells and the tumour microenvironment. The negative impact of fluoride on the central nervous system in children and the growing incidence of pediatric brain tumours since the mid-1980s should serve as the most powerful motivations in our efforts to explain this phenomenon.

Fluorine is a trace element which has not received much attention in basic and clinical research. Nonetheless, with each passing year, there are more and more new papers shedding new light on its still unknown pleiotropic effects. The latest reports from studies on BBB permeation and the effects of fluoride on brain metabolism should inspire researchers to work toward a better understanding of its mechanisms of action. There have now been several studies on the role of micro- and macroelements in the development and treatment of gliomas and, surprisingly, in each one, fluoride has been completely overlooked in the analysis of minerals in brain tumours and whole brains.

## Data Availability

No data was used for the research described in the article.

## Abbreviations

**AKT**—protein kinase B; **AMP**—adenosine-3’, 5’-monophosphate; **AMPA**—α-amino-3-hydroxy-5-methyl-4-isoxazole propionic acid; **AMPA-R**—AMPA receptor; **Atg**—autophagy related proteins family; **ATM**—ataxia telangiectasia mutated; **BBB**—blood–brain barrier; **Bcl-2**—B-cell CLL/lymphoma 2; **CA1**—*cornu ammonis*; **CAT**—catalase; **CNS**—central nervous system; **COXs**—cyclooxygenase; **CSF**—cerebro-spinal fluid; **DG**—*gyrus dentatus*; **Dhh**—Desert hedgehog; **Dkk-1**—Dickkopf; **ECM**—extracellular matrix; **EGF**—epidermal growth factor; **EGFR**—epidermal growth factor receptor; **EMT**—epithelial-mesenchymal transition; **ERK**—extracellular signal-regulated kinase; **GBM**—glioblastoma; **GLI**—glioma-associated oncogene; **GluR1**—glutamate receptor subunit 1; **GLUT1**—glucose transporter 1; **GPx**—glutathione peroxidase; **GR**—glutathione reductase; **GSC**—glioma stem cell; **GSH**—glutathione; **GSK-3β**—glycogen synthase kinase 3β; **GSSG**—oxidized form of glutathione; **HH**—Hedgehog proteins family; **HIF-1**—hypoxia-induced factor; **HMGB1**—high mobility group box protein 1; **ICAM-1**—intercellular adhesion molecule 1; **IDH**—isocitrate dehydrogenase; **IFNγ**—interferon γ; **IGF-1R**—insulin-like growth factor receptor; **IGF2**—insulin like growth factor 2; **Ihh**—Indian hedgehog; **IL**—interleukin; **IL-R**—interleukin receptor; **iNOS**—inducible nitric oxide synthase; **InsRs**—insulin receptors; **IQ**—intelligence quotient; **JNK**—c-Jun N-terminal kinases; **KPNA2**—carioferrin α2; **MAPK**—mitogen-activated protein; **MCH-I**—major histocompatibility complex 1; **MCP-1**—monocyte chemotactic protein-1; **MDM2**—E3 ubiquitin protein ligase; **MEK**—mitogen-activated protein kinase kinase; **MGMT**—methyl-guanine methyl transferase gene; **miR, miRNA**—micro RNA; **MMPs**—matrix metalloproteinases; **mPGES1**—microsomal prostaglandin E1 synthase; **mTOR**—mammalian target of rapamycin; **NF-κB**—nuclear factor κB; **NOX**—NADPH oxidase; **Nrf2**—nuclear factor 2; **PGE2**—prostaglandin E2; **PGs**—prostaglandins; **PI3K**—phosphatidylinositol 3-kinase; **PIQ**—non-verbal intelligence; **PKA**—protein kinase type A; **PTCH**—Patched transmembrane protein; **PTEN**—phosphatase and tensin homolog; **RhoA**—Ras homolog family member A; **RNS**—reactive species; **ROS**—reactive oxygen species; **Shh**—Sonic hedgehog; **SMO**—Smoothened; **SNAIL**—Snail family zinc finger; **SOCS**—cytokine signaling suppressors; **SOD**—superoxide dismutase; **Sox9**—Sry- like HMG-box protein 9; **Src**—non-receptor tyrosine kinases; **STAT3**—signal transducers and activators of transcription; **TAC**—total antioxidant capacity; **TGF-β**—transforming growth factor β; **TIMPs**—tissue inhibitors of matrix metalloproteinases; **TMZ**—temozolomide; **TNFα**—tumour necrosis factor α; **uPA**—urokinase-type plasminogen activator; **uPAR**—urokinase plasminogen activator receptor; **VCAM-1**—vascular cell adhesion protein 1; **VDR**—vitamin D receptor; **VEGF**—vascular endothelial growth factor; **VEGFR**—vascular endothelial growth factor receptor; **Wnt**—Wingless/Int1 Trail pathway; **Zeb1**—zinc finger E-box binding homeobox 1.

## References

[1] Gupte A., Mumper R.J. (2009). Elevated copper and oxidative stress in cancer cells as a target for cancer treatment. Cancer Treat. Rev..

[2] Kuo H.W., Chen S.F., Wu C.C., Chen D.R., Lee J.H. (2002). Serum and Tissue Trace Elements in Patients with Breast Cancer in Taiwan. Biol. Trace Elem. Res..

[3] Ostrakhovitch E., Cherian M. (2004). Differential regulation of signal transduction pathways in wild type and mutated p53 breast cancer epithelial cells by copper and zinc. Arch. Biochem. Biophys..

[4] Torti S., Manz D., Paul B., Blanchette-Farra N., Torti F. (2018). Iron and Cancer. Annu. Rev. Nutr..

[5] Coombs M.R.P., Grant T., Greenshields A.L., Arsenault D.J., Holbein B.E., Hoskin D.W. (2015). Inhibitory effect of iron withdrawal by chelation on the growth of human and murine mammary carcinoma and fibrosarcoma cells. Exp. Mol. Pathol..

[6] García J.A., Fernández D.T., Álvarez E.A., González E.B., Montes-Bayón M., Sanz-Medel A. (2016). Iron speciation, ferritin concentrations and Fe: Ferritin ratios in different malignant breast cancer cell lines: On the search for cancer biomarkers. Metallomics.

[7] Stepien M., Jenab M., Freisling H., Becker N.-P., Czuban M., Tjønneland A., Olsen A., Overvad K., Boutron-Ruault M.-C., Mancini F. (2017). Pre-diagnostic copper and zinc biomarkers and colorectal cancer risk in the European Prospective Investigation into Cancer and Nutrition cohort. Carcinogenesis.

[8] Razaghi A., Poorebrahim M., Sarhan D., Björnstedt M. (2021). Selenium stimulates the antitumour immunity: Insights to future research. Eur. J. Cancer.

[9] Serna J., Bergwitz C. (2020). Importance of Dietary Phosphorus for Bone Metabolism and Healthy Aging. Nutrients.

[10] Phipps O., Brookes M., Al-Hassi H. (2021). Iron deficiency, immunology, and colorectal cancer. Nutr. Rev..

[11] Paganoni R., Lechel A., Spasic M.V. (2021). Iron at the Interface of Hepatocellular Carcinoma. Int. J. Mol. Sci..

[12] Cilliers K., Muller C.J.F., Page B.J. (2020). Trace Element Concentration Changes in Brain Tumors: A Review. Anat. Rec..

[13] Mulware S.J. (2013). Comparative Trace Elemental Analysis in Cancerous and Noncancerous Human Tissues Using PIXE. J. Biophys..

[14] Sohrabi M., Gholami A., Azar M.H., Yaghoobi M., Shahi M.M., Shirmardi S., Nikkhah M., Kohi Z., Salehpour D., Khoonsari M.R. (2018). Trace Element and Heavy Metal Levels in Colorectal Cancer: Comparison Between Cancerous and Non-cancerous Tissues. Biol. Trace Elem. Res..

[15] Andrási E., Suhajda M., Sáray I., Bezúr L., Ernyei L., Réffy A. (1993). Concentration of elements in human brain: Glioblastoma multiforme. Sci. Total Environ..

[16] Chandra S., Parker D.J., Barth R.F., Pannullo S.C. (2015). Quantitative imaging of magnesium distribution at single-cell resolution in brain tumors and infiltrating tumor cells with secondary ion mass spectrometry (SIMS). J. Neuro Oncol..

[17] Floriańczyk B., Kaczmarczyk R., Osuchowski J., Trojanowski T. (2007). Metallothionein and manganese concentrations in brain tumors. J. Pre Clin. Clin. Res..

[18] Wandzilak A., Czyzycki M., Radwanska E., Adamek D., Geraki K., Lankosz M. (2015). X-ray fluorescence study of the concentration of selected trace and minor elements in human brain tumours. Spectrochim. Acta Part B At. Spectrosc..

[19] Al-Saleh I., Shinwari N. (2001). Levels of Cadmium, Lead, and Mercury in Human Brain Tumors. Biol. Trace Elem. Res..

[20] Zhuang G., Zhou Y., Lu H., Lu W., Zhou M., Wang Y., Tan M. (1996). Concentration of rare earth elements, As, and Th in human brain and brain tumors, determined by neutron activation analysis. Biol. Trace Elem. Res..

[21] Guth S., Hüser S., Roth A., Degen G., Diel P., Edlund K., Eisenbrand G., Engel K.-H., Epe B., Grune T. (2020). Toxicity of fluoride: Critical evaluation of evidence for human developmental neurotoxicity in epidemiological studies, animal experiments and in vitro analyses. Arch. Toxicol..

[22] Strazielle N., Ghersi-Egea J.-F. (2013). Physiology of Blood–Brain Interfaces in Relation to Brain Disposition of Small Compounds and Macromolecules. Mol. Pharm..

[23] Ghosh D., Ghosh S. (2020). Flouride and Brain: A Review. Int. J. Pharm. Sci. Res..

[24] Grandjean P. (2019). Developmental fluoride neurotoxicity: An updated review. Environ. Health.

[25] Ostrom Q.T., Gittleman H., Truitt G., Boscia A., Kruchko C., Barnholtz-Sloan J.S. (2018). CBTRUS Statistical Report: Primary Brain and Other Central Nervous System Tumors Diagnosed in the United States in 2011–2015. Neuro Oncol..

[26] Ostrom Q.T., Gittleman H., de Blank P.M., Finlay J.L., Gurney J.G., McKean-Cowdin R., Stearns D.S., Wolff J.E., Liu M., Wolinsky Y. (2016). American Brain Tumor Association Adolescent and Young Adult Primary Brain and Central Nervous System Tumors Diagnosed in the United States in 2008–2012. Neuro Oncol..

[27] World Health Organization (2021). CureAll Framework: WHO Global Initiative for Childhood Cancer: Increasing Access, Advancing Quality, Saving Lives.

[28] Patel S., Bhatnagar A., Wear C., Osiro S., Gabriel A., Kimball D., John A., Fields P.J., Tubbs R.S., Loukas M. (2014). Are pediatric brain tumors on the rise in the USA? Significant incidence and survival findings from the SEER database analysis. Childs Nerv. Syst..

[29] Nakamoto T., Rawls H.R. (2018). Fluoride Exposure in Early Life as the Possible Root Cause of Disease In Later Life. J. Clin. Pediatr. Dent..

[30] Raaschou-Nielsen O., Sørensen M., Carstensen H., Jensen T., Bernhardtsen T., Gjerris F., Schmiegelow K. (2006). Increasing incidence of childhood tumours of the central nervous system in Denmark, 1980–1996. Br. J. Cancer.

[31] Smith M.A., Freidlin B., Ries L.A.G., Simon R. (1998). Trends in Reported Incidence of Primary Malignant Brain Tumors in Children in the United States. Gynecol. Oncol..

[32] Jha S., Mishra V., Sharma D., Damodaran T., Whitacre D. (2011). Fluoride in the Environment and Its Metabolism in Humans. Reviews of Environmental Contamination and Toxicology Volume 211.

[33] O’Mullane D.M., Baez R.J., Jones S., Lennon M.A., E Petersen P., Rugg-Gunn A.J., Whelton H., Whitford G.M. (2016). Fluoride and Oral Health. Community Dent Health.

[34] World Health Organization (2019). Preventing Disease through Healthy Environments: Inadequate or Excess Fluoride: A Major Public Health Concern.

[35] Vithanage M., Bhattacharya P. (2015). Fluoride in the environment: Sources, distribution and defluoridation. Environ. Chem. Lett..

[36] Bombik E., Bombik A., Rymuza K. (2020). The influence of environmental pollution with fluorine compounds on the level of fluoride in soil, feed and eggs of laying hens in Central Pomerania, Poland. Environ. Monit. Assess..

[37] Ghanbarian M., Ghanbarian M., Tabatabaie T., Ghanbarian M., Ghadiri S.-K. (2021). Distributing and assessing fluoride health risk in urban drinking water resources in Fars Province, Iran, using the geographical information system. Environ. Geochem. Health.

[38] Jaudenes J.R., Gutiérrez J., Paz S., Rubio C., Hardisson A. (2020). Fluoride Risk Assessment from Consumption of Different Foods Commercialized in a European Region. Appl. Sci..

[39] Riddell J., Malin A., McCague H., Flora D., Till C. (2021). Urinary Fluoride Levels among Canadians with and without Community Water Fluoridation. Int. J. Environ. Res. Public Health.

[40] Strunecka A., Strunecky O. (2020). Mechanisms of Fluoride Toxicity: From Enzymes to Underlying Integrative Networks. Appl. Sci..

[41] Medjedovic E., Medjedovic S., Deljo D., Sukalo A. (2015). Impact of Fluoride on Dental Health Quality. Mater. Socio Medica.

[42] Chen L., Kuang P., Liu H., Wei Q., Cui H., Fang J., Zuo Z., Deng J., Li Y., Wang X. (2019). Sodium Fluoride (NaF) Induces Inflammatory Responses Via Activating MAPKs/NF-κB Signaling Pathway and Reducing Anti-inflammatory Cytokine Expression in the Mouse Liver. Biol. Trace Elem. Res..

[43] Refsnes M., Skuland T., Schwarze P., Lag M., Ovrevik J. (2014). Differential NF-κB and MAPK activation underlies fluoride- and TPA-mediated CXCL8 (IL-8) induction in lung epithelial cells. JIR.

[44] Pan X., Yan W., Qiu B., Liao Y., Liao Y., Wu S., Ming J., Zhang A. (2019). Aberrant DNA methylation of Cyclind-CDK4-p21 is associated with chronic fluoride poisoning. Chem. Interact..

[45] Aulestia F.J., Groeling J., Bomfim G.H.S., Costiniti V., Manikandan V., Chaloemtoem A., Concepcion A.R., Li Y., Ii L.E.W., Idaghdour Y. (2020). Fluoride exposure alters Ca^2+^ signaling and mitochondrial function in enamel cells. Sci. Signal..

[46] Nagendra A.H., Bose B., Shenoy P.S. (2021). Recent advances in cellular effects of fluoride: An update on its signalling pathway and targeted therapeutic approaches. Mol. Biol. Rep..

[47] Jianjie C., Wenjuan X., Jinling C., Jie S., Ruhui J., Meiyan L. (2016). Fluoride caused thyroid endocrine disruption in male zebrafish (Danio rerio). Aquat. Toxicol..

[48] Cao J., Chen Y., Chen J., Yan H., Li M., Wang J. (2016). Fluoride exposure changed the structure and the expressions of Y chromosome related genes in testes of mice. Chemosphere.

[49] Han H., Sun Z., Luo G., Wang C., Wei R., Wang J. (2015). Fluoride exposure changed the structure and the expressions of reproductive related genes in the hypothalamus–pituitary–testicular axis of male mice. Chemosphere.

[50] Iano F.G., Ferreira M.C., Quaggio G.B., Fernandes M.S., Oliveira R.C., Ximenes V.F., Buzalaf M.A.R. (2014). Effects of chronic fluoride intake on the antioxidant systems of the liver and kidney in rats. J. Fluor. Chem..

[51] Liu H., Gao Y., Sun L., Li M., Li B., Sun D. (2014). Assessment of relationship on excess fluoride intake from drinking water and carotid atherosclerosis development in adults in fluoride endemic areas, China. Int. J. Hyg. Environ. Health.

[52] Liu P., Li R., Tian X., Zhao Y., Li M., Wang M., Ying X., Yuan J., Xie J., Yan X. (2021). Co-exposure to fluoride and arsenic disrupts intestinal flora balance and induces testicular autophagy in offspring rats. Ecotoxicol. Environ. Saf..

[53] Raina R., Baba N.A., Verma P.K., Sultana M., Singh M. (2015). Hepatotoxicity Induced by Subchronic Exposure of Fluoride and Chlorpyrifos in Wistar Rats: Mitigating Effect of Ascorbic Acid. Biol. Trace Elem. Res..

[54] Song C., Fu B., Zhang J., Zhao J., Yuan M., Peng W., Zhang Y., Wu H. (2017). Sodium fluoride induces nephrotoxicity via oxidative stress-regulated mitochondrial SIRT3 signaling pathway. Sci. Rep..

[55] Liu Y.-J., Gao Q., Wu C.-X., Guan Z.-Z. (2010). Alterations of nAChRs and ERK1/2 in the brains of rats with chronic fluorosis and their connections with the decreased capacity of learning and memory. Toxicol. Lett..

[56] Reddy P., Reddy K., Kumar K. (2011). Neurodegenerative Changes in Different Regions of Brain, Spinal Cord and Sciatic Nerve of Rats Treated with Sodium Fluoride. J. Med. Allied Sci..

[57] Wu C., Gu X., Ge Y., Jianhai Z., Wang J. (2006). Effects of high fluoride and arsenic on brain biochemical indexes and learning-memory in rats. Fluoride.

[58] Qing-Feng S., Ying-Peng X., Tian-Tong X. (2019). Matrix metalloproteinase-9 and p53 involved in chronic fluorosis induced blood-brain barrier damage and neurocyte changes. Arch. Med. Sci..

[59] Opydo J., Borysewicz-Lewickaa M. (2007). Transplacental passage of fluoride in pregnant Polish women assessed on the basis of fluoride concentrations in maternal and cord blood plasma. Fluoride.

[60] Niu Q., Chen J., Xia T., Li P., Zhou G., Xu C., Zhao Q., Dong L., Zhang S., Wang A. (2018). Excessive ER stress and the resulting autophagic flux dysfunction contribute to fluoride-induced neurotoxicity. Environ. Pollut..

[61] Bartos M., Gumilar F., Gallegos C.E., Bras C., Dominguez S., Cancela L.M., Minetti A. (2019). Effects of Perinatal Fluoride Exposure on Short- and Long-Term Memory, Brain Antioxidant Status, and Glutamate Metabolism of Young Rat Pups. Int. J. Toxicol..

[62] Kupnicka P., Listos J., Tarnowski M., Kolasa-Wołosiuk A., Wąsik A., Łukomska A., Barczak K., Gutowska I., Chlubek D., Baranowska-Bosiacka I. (2020). Fluoride Affects Dopamine Metabolism and Causes Changes in the Expression of Dopamine Receptors (D1R and D2R) in Chosen Brain Structures of Morphine-Dependent Rats. Int. J. Mol. Sci..

[63] Sun Z., Zhang Y., Xue X., Niu R., Wang J. (2018). Maternal fluoride exposure during gestation and lactation decreased learning and memory ability, and glutamate receptor mRNA expressions of mouse pups. Hum. Exp. Toxicol..

[64] Lopes G.O., Ferreira M.K.M., Davis L., Bittencourt L.O., Aragão W.A.B., Dionizio A., Buzalaf M.A.R., Crespo-Lopez M.E., Maia C.S.F., Lima R.R. (2020). Effects of Fluoride Long-Term Exposure over the Cerebellum: Global Proteomic Profile, Oxidative Biochemistry, Cell Density, and Motor Behavior Evaluation. Int. J. Mol. Sci..

[65] Jiang C., Zhang S., Liu H., Guan Z., Zeng Q., Zhang C., Lei R., Xia T., Wang Z., Yang L. (2014). Low Glucose Utilization and Neurodegenerative Changes Caused by Sodium Fluoride Exposure in Rat’s Developmental Brain. Neuromol. Med..

[66] Rogalska A., Kuter K., Żelazko A., Głogowska-Gruszka A., Świętochowska E., Nowak P. (2017). Fluoride Alteration of [3H]Glucose Uptake in Wistar Rat Brain and Peripheral Tissues. Neurotox. Res..

[67] Guan Z.-Z., Wang Y.-N., Xiao K.-Q., Dai D.-Y., Chen Y.-H., Liu J.-L., Sindelar P., Dallner G. (1998). Influence of Chronic Fluorosis on Membrane Lipids in Rat Brain. Neurotoxicol. Teratol..

[68] Adedara I., Olabiyi B., Ojuade T., Idris U., Onibiyo E., Farombi E. (2017). Taurine reverses sodium fluoride-mediated increase in inflammation, caspase-3 activity, and oxidative damage along the brain-pituitary-gonadal axis in male rats. Can. J. Physiol. Pharmacol..

[69] Dec K., Łukomska A., Skonieczna-Żydecka K., Jakubczyk K., Tarnowski M., Lubkowska A., Baranowska-Bosiacka I., Styburski D., Skórka-Majewicz M., Maciejewska D. (2020). Chronic Exposure to Fluoride Affects GSH Level and NOX4 Expression in Rat Model of This Element of Neurotoxicity. Biomolecules.

[70] Shuhua X., Ziyou L., Ling Y., Fei W., Sun G. (2012). A Role of Fluoride on Free Radical Generation and Oxidative Stress in BV-2 Microglia Cells. Mediat. Inflamm..

[71] Yan N., Liu Y., Liu S., Cao S., Wang F., Wang Z., Xi S. (2016). Fluoride-Induced Neuron Apoptosis and Expressions of Inflammatory Factors by Activating Microglia in Rat Brain. Mol. Neurobiol..

[72] Liu X.-L., Li C.-C., Liu K.-J., Cui C.-Y., Zhang Y.-Z., Liu Y. (2012). The Influence of Fluoride on the Expression of Inhibitors of Wnt/β-Catenin Signaling Pathway in Rat Skin Fibroblast Cells. Biol. Trace Elem. Res..

[73] Xu B., Xu Z., Xia T., He P., Gao P., He W., Zhang M., Guo L., Niu Q., Wang A. (2011). Effects of the Fas/Fas-L pathway on fluoride-induced apoptosis in SH-SY5Y cells. Environ. Toxicol..

[74] Tu W., Zhang Q., Liu Y., Han L., Wang Q., Chen P., Zhang S., Wang A., Zhou X. (2018). Fluoride induces apoptosis via inhibiting SIRT1 activity to activate mitochondrial p53 pathway in human neuroblastoma SH-SY5Y cells. Toxicol. Appl. Pharmacol..

[75] Zhou G., Tang S., Yang L., Niu Q., Chen J., Xia T., Wang S., Wang M., Zhao Q., Liu L. (2019). Effects of long-term fluoride exposure on cognitive ability and the underlying mechanisms: Role of autophagy and its association with apoptosis. Toxicol. Appl. Pharmacol..

[76] Bashash M., Thomas D., Hu H., Martinez-Mier E.A., Sanchez B., Basu N., Peterson K., Ettinger A., Wright R., Zhang Z. (2017). Prenatal Fluoride Exposure and Cognitive Outcomes in Children at 4 and 6–12 Years of Age in Mexico. Environ. Health Perspect..

[77] Cao K., Xiang J., Dong Y.-T., Xu Y., Li Y., Song H., Zeng X.-X., Ran L.-Y., Hong W., Guan Z.-Z. (2019). Exposure to fluoride aggravates the impairment in learning and memory and neuropathological lesions in mice carrying the APP/PS1 double-transgenic mutation. Alzheimer’s Res. Ther..

[78] Farmus L., Till C., Green R., Hornung R., Mier E.A.M., Ayotte P., Muckle G., Lanphear B.P., Flora D.B. (2021). Critical windows of fluoride neurotoxicity in Canadian children. Environ. Res..

[79] Green R., Lanphear B., Hornung R., Flora D., Martinez-Mier E.A., Neufeld R., Ayotte P., Muckle G., Till C. (2019). Association Between Maternal Fluoride Exposure During Pregnancy and IQ Scores in Offspring in Canada. JAMA Pediatr..

[80] Russ T.C., Killin L.O.J., Hannah J., Batty G., Deary I.J., Starr J.M. (2020). Aluminium and fluoride in drinking water in relation to later dementia risk. Br. J. Psychiatry.

[81] Malin A.J., Till C. (2015). Exposure to fluoridated water and attention deficit hyperactivity disorder prevalence among children and adolescents in the United States: An ecological association. Environ. Health.

[82] Agalakova N.I., Nadei O. (2020). Inorganic fluoride and functions of brain. Crit. Rev. Toxicol..

[83] Broadbent J.M., Thomson W.M., Ramrakha S., Moffitt T.E., Zeng J., Page L.A.F., Poulton R. (2015). Community Water Fluoridation and Intelligence: Prospective Study in New Zealand. Am. J. Public Health.

[84] Saeed M., Malik R.N., Kamal A. (2020). Fluorosis and cognitive development among children (6–14 years of age) in the endemic areas of the world: A review and critical analysis. Environ. Sci. Pollut. Res..

[85] Till C., Green R. (2020). Controversy: The evolving science of fluoride: When new evidence doesn’t conform with existing beliefs. Pediatr. Res..

[86] Habib A., Hoppe M., Beiriger J., Kodavali C.V., Edwards L., Zinn P.O. (2022). Letter: Glioblastoma Cell of Origin. Stem Cell Rev. Rep..

[87] Claus E.B., Walsh K.M., Wiencke J.K., Molinaro A.M., Wiemels J.L., Schildkraut J.M., Bondy M.L., Berger M., Jenkins R., Wrensch M. (2015). Survival and low-grade glioma: The emergence of genetic information. Neurosurg. Focus.

[88] Kanderi T., Gupta V. (2021). Glioblastoma Multiforme. StatPearls.

[89] Stupp R., Mason W., van den Bent M., Weller M., Fisher B., Taphoorn M., Belanger K., Brandes A., Marosi C., Bogdahn U. (2005). Radiotherapy plus concomitant and adjuvant temozolomide for glioblastoma. N. Engl. J. Med..

[90] Arora A., Somasundaram K. (2019). Glioblastoma vs temozolomide: Can the red queen race be won?. Cancer Biol. Ther..

[91] Alfonso J.C.L., Talkenberger K., Seifert M., Klink B., Hawkins-Daarud A., Swanson K.R., Hatzikirou H., Deutsch A. (2017). The biology and mathematical modelling of glioma invasion: A review. J. R. Soc. Interface.

[92] Katsuno Y., Lamouille S., Derynck R. (2013). TGF-β signaling and epithelial–mesenchymal transition in cancer progression. Curr. Opin. Oncol..

[93] Manini I., Caponnetto F., Bartolini A., Ius T., Mariuzzi L., Di Loreto C., Beltrami A., Cesselli D. (2018). Role of Microenvironment in Glioma Invasion: What We Learned from In Vitro Models. Int. J. Mol. Sci..

[94] Koh I., Cha J., Park J., Choi J., Kang S.-G., Kim P. (2018). The mode and dynamics of glioblastoma cell invasion into a decellularized tissue-derived extracellular matrix-based three-dimensional tumor model. Sci. Rep..

[95] Paw I., Carpenter R.C., Watabe K., Debinski W., Lo H.-W. (2015). Mechanisms regulating glioma invasion. Cancer Lett..

[96] Becker A.P., Sells B.E., Haque S.J., Chakravarti A. (2021). Tumor Heterogeneity in Glioblastomas: From Light Microscopy to Molecular Pathology. Cancers.

[97] Inda M.-d.-M., Bonavia R., Seoane J. (2014). Glioblastoma Multiforme: A Look Inside Its Heterogeneous Nature. Cancers.

[98] Patel A.P., Tirosh I., Trombetta J.J., Shalek A.K., Gillespie S.M., Wakimoto H., Cahill D.P., Nahed B.V., Curry W.T., Martuza R.L. (2014). Single-cell RNA-seq highlights intratumoral heterogeneity in primary glioblastoma. Science.

[99] Singh N., Miner A., Hennis L., Mittal S. (2021). Mechanisms of temozolomide resistance in glioblastoma—A comprehensive review. Cancer Drug Resist..

[100] Feldheim J., Kessler A.F., Monoranu C.M., Ernestus R.-I., Löhr M., Hagemann C. (2019). Changes of O6-Methylguanine DNA Methyltransferase (MGMT) Promoter Methylation in Glioblastoma Relapse—A Meta-Analysis Type Literature Review. Cancers.

[101] Wang K., Pan L., Che X., Cui D., Li C. (2010). Sonic Hedgehog/GLI1 signaling pathway inhibition restricts cell migration and invasion in human gliomas. Neurol. Res..

[102] Avci N., Ebrahimzadeh-Pustchi S., Akay Y., Esquenazi Y., Tandon N., Zhu J.-J., Akay M. (2020). NF-κB inhibitor with Temozolomide results in significant apoptosis in glioblastoma via the NF-κB(p65) and actin cytoskeleton regulatory pathways. Sci. Rep..

[103] Latour M., Her N.-G., Kesari S., Nurmemmedov E. (2021). WNT Signaling as a Therapeutic Target for Glioblastoma. Int. J. Mol. Sci..

[104] D’Amico M., De Amicis F. (2022). Aberrant Notch signaling in gliomas: A potential landscape of actionable converging targets for combination approach in therapies resistance. Cancer Drug Resist..

[105] Choudhry Z., Rikani A.A., Choudhry A.M., Tariq S., Zakaria F., Asghar M.W., Sarfraz M., Haider K., Shafiq A.A., Mobassarah N.J. (2014). Sonic hedgehog signalling pathway: A complex network. Ann. Neurosci..

[106] Carpenter R., Lo H.-W. (2012). Hedgehog pathway and GLI1 isoforms in human cancer. Discov. Med..

[107] Zhu H., Lo H.-W. (2010). The Human Glioma-Associated Oncogene Homolog 1 (GLI1) Family of Transcription Factors in Gene Regulation and Diseases. Curr. Genom..

[108] Pietrobono S., Gagliardi S., Stecca B. (2019). Non-canonical Hedgehog Signaling Pathway in Cancer: Activation of GLI Transcription Factors Beyond Smoothened. Front. Genet..

[109] Xie J., Aszterbaum M., Zhang X., Bonifas J., Zachary C., Epstein E., McCormick F. (2001). A role of PDGFRalpha in basal cell carcinoma proliferation. Proc. Natl. Acad. Sci. USA.

[110] Zhu H., Carpenter R.L., Han W., Lo H.-W. (2014). The GLI1 splice variant TGLI1 promotes glioblastoma angiogenesis and growth. Cancer Lett..

[111] Doheny D., Sirkisoon S., Carpenter R., Aguayo N., Regua A., Anguelov M., Manore S., Arrigo A., Jalboush S., Wong G. (2020). Combined inhibition of JAK2-STAT3 and SMO-GLI1/tGLI1 pathways suppresses breast cancer stem cells, tumor growth, and metastasis. Oncogene.

[112] Sirkisoon S.R., Carpenter R.L., Rimkus T., Doheny D., Zhu D., Aguayo N.R., Xing F., Chan M., Ruiz J., Metheny-Barlow L.J. (2020). TGLI1 transcription factor mediates breast cancer brain metastasis via activating metastasis-initiating cancer stem cells and astrocytes in the tumor microenvironment. Oncogene.

[113] Wang K., Chen D., Qian Z., Cui D., Gao L., Lou M. (2017). Hedgehog/Gli1 signaling pathway regulates MGMT expression and chemoresistance to temozolomide in human glioblastoma. Cancer Cell Int..

[114] Li J., Cai J., Zhao S., Yao K., Sun Y., Li Y., Chen L., Li R., Zhai X., Zhang J. (2016). GANT61, a GLI inhibitor, sensitizes glioma cells to the temozolomide treatment. J. Exp. Clin. Cancer Res..

[115] Ji M., Wang L., Chen J., Xue N., Wang C., Lai F., Wang R., Yu S., Jin J., Chen X. (2018). CAT_3_, a prodrug of 13a(S)-3-hydroxyl-6,7-dimethoxyphenanthro[9,10-b]-indolizidine, circumvents temozolomide-resistant glioblastoma via the Hedgehog signaling pathway, independently of O^6^-methylguanine DNA methyltransferase expression. Onco Targets Ther..

[116] Wang Y., Zhang X., Zhao Z., Xu H. (2017). Preliminary Analysis of MicroRNAs Expression Profiling in MC3T3-E1 Cells Exposed to Fluoride. Biol. Trace Elem. Res..

[117] Zhao L., Yu Y., Deng C. (2014). Protein and mRNA expression of Shh, Smo and Gli1 and inhibition by cyclopamine in hepatocytes of rats with chronic fluorosis. Toxicol. Lett..

[118] Zhu Z., Yu Y., Chen R. (2018). Role of hedgehog signaling pathway on cartilage tissue damage in chronic fluorosis rats. Chin. J. Public Health.

[119] Deng C., Xu L., Zhang Y., Zhao L., Linghu Y., Yu Y. (2021). The value of the hedgehog signal in osteoblasts in fluoride-induced bone-tissue injury. J. Orthop. Surg. Res..

[120] Sun S.-C. (2011). Non-canonical NF-κB signaling pathway. Cell Res..

[121] Bhat K., Balasubramaniyan V., Vaillant B., Ezhilarasan R., Hummelink K., Hollingsworth F., Wani K., Heathcock L., James J., Goodman L. (2013). Mesenchymal Differentiation Mediated by NF-κB Promotes Radiation Resistance in Glioblastoma. Cancer Cell.

[122] Friedmann-Morvinski D., Narasimamurthy R., Xia Y., Myskiw C., Soda Y., Verma I. (2016). Targeting NF-κB in glioblastoma: A therapeutic approach. Sci. Adv..

[123] Gray G., McFarland B., Nozell S., Benveniste E. (2014). NF-κB and STAT3 in glioblastoma: Therapeutic targets coming of age. Expert Rev. Neurother..

[124] McFarland B., Gray G., Nozell S., Hong S., Benveniste E. (2013). Activation of the NF-κB Pathway by the STAT3 Inhibitor JSI-124 in Human Glioblastoma Cells. Mol. Cancer Res..

[125] Raychaudhuri B., Han Y., Lu T., Vogelbaum M. (2007). Aberrant constitutive activation of nuclear factor kappaB in glioblastoma multiforme drives invasive phenotype. J. Neuro Oncol..

[126] Fianco G., Mongiardi M., Levi A., De Luca T., Desideri M., Trisciuoglio D., Del Bufalo D., Cinà I., Di Benedetto A., Mottolese M. (2017). Caspase-8 contributes to angiogenesis and chemotherapy resistance in glioblastoma. Elife.

[127] Jiang L., Song L., Wu J., Yang Y., Zhu X., Hu B., Cheng S.-Y., Li M. (2013). Bmi-1 promotes glioma angiogenesis by activating NF-κB signaling. PLoS ONE.

[128] Ritchie C., Giordano A., Khalili K. (2000). Integrin involvement in glioblastoma multiforme: Possible regulation by NF-kappaB. J. Cell Physiol..

[129] Zhang J., Zhu Y., Shi Y., Han Y., Liang C., Feng Z., Zheng H., Eng M., Wang J. (2017). Fluoride-Induced Autophagy via the Regulation of Phosphorylation of Mammalian Targets of Rapamycin in Mice Leydig Cells. J. Agric. Food Chem..

[130] Zhang J.-F., Wang P., Yan Y.-J., Li Y., Guan M.-W., Yu J.-J., Wang X.-D. (2017). IL-33 enhances glioma cell migration and invasion by upregulation of MMP2 and MMP9 via the ST2-NF-κB pathway. Oncol. Rep..

[131] Liu T., Ma W., Xu H., Huang M., Zhang D., He Z., Zhang L., Brem S., O’Rourke D., Gong Y. (2018). PDGF-mediated mesenchymal transformation renders endothelial resistance to anti-VEGF treatment in glioblastoma. Nat. Commun..

[132] Yamini B. (2018). NF-κB, Mesenchymal Differentiation and Glioblastoma. Cells.

[133] Soubannier V., Stifani S. (2017). NF-κB Signalling in Glioblastoma. Biomedicines.

[134] Kim Y., Varn F.S., Park S.-H., Yoon B.W., Park H.R., Lee C., Verhaak R.G.W., Paek S.H. (2021). Perspective of mesenchymal transformation in glioblastoma. Acta Neuropathol. Commun..

[135] Yu X., Wang M., Zuo J., Wahafu A., Mao P., Li R., Wu W., Xie W., Wang J. (2019). Nuclear factor I A promotes temozolomide resistance in glioblastoma via activation of nuclear factor κB pathway. Life Sci..

[136] Wang X., JIia L., Jin X., Liu Q., Cao W., Gao X., Yang M., Sun B. (2015). NF-κB inhibitor reverses temozolomide resistance in human glioma TR/U251 cells. Oncol. Lett..

[137] Nogueira L., Ruiz-Ontañon P., Vazquez-Barquero A., Moris F., Fernandez-Luna J.L. (2011). The NFκB pathway: A therapeutic target in glioblastoma. Oncotarget.

[138] Stachowska E., Baśkiewicz-Masiuk E., Machaliński B., Rybicka M., Gutowska I., Bober J., Grymula K., Dziedziejko V., Chlubek D. (2005). Sodium Fluoride Enhancement Of Monocyte Differentiation Via Nuclear Factor Κb Mechanism. Fluoride.

[139] Tian Y., Huo M., Li G., Li Y., Wang J. (2016). Regulation of LPS-induced mRNA expression of pro-inflammatory cytokines via alteration of NF-κB activity in mouse peritoneal macrophages exposed to fluoride. Chemosphere.

[140] Chen Q., Wang Z., Xiong Y., Zou X., Liu Z. (2010). Comparative study of p38 MAPK signal transduction pathway of peripheral blood mononuclear cells from patients with coal-combustion-type fluorosis with and without high hair selenium levels. Int. J. Hyg. Environ. Health.

[141] Luo Q., Cui H., Deng H., Kuang P., Liu H., Lu Y., Fang J., Zuo Z., Deng J., Li Y. (2017). Sodium fluoride induces renal inflammatory responses by activating NF-κB signaling pathway and reducing anti-inflammatory cytokine expression in mice. Oncotarget.

[142] Deng H., Kuang P., Cui H., Luo Q., Liu H., Lu Y., Fang J., Zuo Z., Deng J., Li Y. (2017). Sodium fluoride induces apoptosis in mouse splenocytes by activating ROS-dependent NF-κB signaling. Oncotarget.

[143] Sana S., Ghosh S., Das N., Sarkar S., Mandal A. (2017). Vesicular melatonin efficiently downregulates sodium fluoride-induced rat hepato- and broncho-TNF-α, TGF-β expressions, and associated oxidative injury: A comparative study of liposomal and nanoencapsulated forms. Int. J. Nanomed..

[144] Tiwari S., Gupta S.K., Kumar K., Trivedi R., Godbole M.M. (2004). Simultaneous Exposure of Excess Fluoride and Calcium Deficiency Alters VDR, CaR, and Calbindin D 9 k mRNA Levels in Rat Duodenal Mucosa. Calcif. Tissue Int..

[145] Sun J., Kong J., Duan Y., Szeto F., Liao A., Madara J., Li Y. (2006). Increased NF-kappaB activity in fibroblasts lacking the vitamin D receptor. Am. J. Physiol. Endocrinol. Metab..

[146] Łukomska A., Baranowska-Bosiacka I., Dec K., Pilutin A., Tarnowski M., Jakubczyk K., Żwierełło W., Skórka-Majewicz M., Chlubek D., Gutowska I. (2020). Changes in Gene and Protein Expression of Metalloproteinase-2 and -9 and their Inhibitors TIMP2 and TIMP3 in Different Parts of Fluoride-Exposed Rat Brain. Int. J. Mol. Sci..

[147] Zhang J., Zhu W.-J., Xu X.-H., Zhang Z.-G. (2011). Effect of fluoride on calcium ion concentration and expression of nuclear transcription factor kappa-B ρ65 in rat hippocampus. Exp. Toxicol. Pathol..

[148] Zhang M., Wang A., Xia T., He P. (2008). Effects of fluoride on DNA damage, S-phase cell-cycle arrest and the expression of NF-kappaB in primary cultured rat hippocampal neurons. Toxicol. Lett..

[149] Zhang X., Chen T., Zhang J., Mao Q., Li S., Xiong W., Qiu Y., Xie Q., Ge J. (2012). Notch1 promotes glioma cell migration and invasion by stimulating β-catenin and NF-κB signaling via AKT activation. Cancer Sci..

[150] Lee Y., Lee J.-K., Ahn S.H., Lee J., Nam D.-H. (2016). WNT signaling in glioblastoma and therapeutic opportunities. Lab. Investig..

[151] Kahlert U.D., Maciaczyk D., Doostkam S., Orr B.A., Simons B., Bogiel T., Reithmeier T., Prinz M., Schubert J., Niedermann G. (2012). Activation of canonical WNT/β-catenin signaling enhances in vitro motility of glioblastoma cells by activation of ZEB1 and other activators of epithelial-to-mesenchymal transition. Cancer Lett..

[152] Basu S., Cheriyamundath S., Ben-Ze’Ev A. (2018). Cell–cell adhesion: Linking Wnt/β-catenin signaling with partial EMT and stemness traits in tumorigenesis. F1000Research.

[153] Zhang M., Atkinson R.L., Rosen J.M. (2010). Selective targeting of radiation-resistant tumor-initiating cells. Proc. Natl. Acad. Sci. USA.

[154] Semenov M.V., Habas R., MacDonald B.T., He X. (2007). SnapShot: Noncanonical Wnt Signaling Pathways. Cell.

[155] Rao T., Kühl M. (2010). An updated overview on Wnt signaling pathways: A prelude for more. Circ. Res..

[156] Binda E., Visioli A., Giani F., Trivieri N., Palumbo O., Restelli S., Dezi F., Mazza T., Fusilli C., Legnani F. (2017). Wnt5a Drives an Invasive Phenotype in Human Glioblastoma Stem-like Cells. Cancer Res..

[157] Kamino M., Kishida M., Kibe T., Ikoma K., Iijima M., Hirano H., Tokudome M., Koriyama C., Kishida S., Chen L. (2011). Wnt-5a signaling is correlated with infiltrative activity in human glioma by inducing cellular migration and MMP-2. Cancer Sci..

[158] Wickström M., Dyberg C., Milosevic J., Einvik C., Calero R., Sveinbjörnsson B., Sandén E., Darabi A., Siesjö P., Kool M. (2015). Wnt/β-catenin pathway regulates MGMT gene expression in cancer and inhibition of Wnt signalling prevents chemoresistance. Nat. Commun..

[159] Chen R., Zhao L.-D., Liu H., Li H.-H., Ren C., Zhang P., Guo K.-T., Zhang H.-X., Geng D.-Q., Zhang C.-Y. (2017). Fluoride Induces Neuroinflammation and Alters Wnt Signaling Pathway in BV2 Microglial Cells. Inflammation.

[160] Zeng Q.B., Xu Y.Y., Tu C.L., Yu X., Yang J., Hong F. (2019). [Biological exposure limits caused by co exposure to fluoride and arsenic based on Wnt signaling pathway]. Ying Yong Sheng Tai Xue Bao.

[161] Luo K., Qin Y., Ouyang T., Wang X., Zhang A., Luo P., Pan X. (2021). let-7c-5p regulates CyclinD1 in fluoride-mediated osteoblast proliferation and activation. Toxicol. Sci..

[162] Peng L., Li Y., Shusterman K., Kuehl M., Gibson C. (2011). Wnt-RhoA signaling is involved in dental enamel development. Eur. J. Oral Sci..

[163] Shusterman K., Gibson C., Li Y., Healey M., Peng L. (2014). Wnt-RhoA Signaling Pathways in Fluoride-Treated Ameloblast-Lineage Cells. CTO.

[164] Nadei O.V., Khvorova I.A., Agalakova N.I. (2020). Cognitive Decline of Rats with Chronic Fluorosis Is Associated with Alterations in Hippocampal Calpain Signaling. Biol. Trace Elem. Res..

[165] Mathieu P., Adami P.V.M., Morelli L. (2013). Notch signaling in the pathologic adult brain. Biomol. Concepts.

[166] Takebe N., Nguyen D., Yang S.X. (2013). Targeting Notch signaling pathway in cancer: Clinical development advances and challenges. Pharmacol. Ther..

[167] Kanamori M., Kawaguchi T., Nigro J.M., Feuerstein B.G., Berger M.S., Miele L., Pieper R.O. (2007). Contribution of Notch signaling activation to human glioblastoma multiforme. J. Neurosurg..

[168] Biswas S., Rao C.M. (2017). Epigenetics in cancer: Fundamentals and Beyond. Pharmacol. Ther..

[169] Yi G.-Z., Huang G., Guo M., Zhang X., Wang H., Deng S., Li Y., Xiang W., Chen Z., Pan J. (2019). Acquired temozolomide resistance in MGMT-deficient glioblastoma cells is associated with regulation of DNA repair by DHC2. Brain.

[170] Bazzoni R., Bentivegna A. (2019). Role of Notch Signaling Pathway in Glioblastoma Pathogenesis. Cancers.

[171] Han N., Hu G., Shi L., Long G., Yang L., Xi Q., Guo Q., Wang J., Dong Z., Zhang M. (2017). *Notch1*ablation radiosensitizes glioblastoma cells. Oncotarget.

[172] Xing Z.-Y., Sun L.-G., Guo W.-J. (2015). Elevated expression of Notch-1 and EGFR induced apoptosis in glioblastoma multiforme patients. Clin. Neurol. Neurosurg..

[173] Qiao L., Liu X., He Y., Zhang J., Huang H., Bian W., Chilufya M.M., Zhao Y., Han J. (2021). Progress of Signaling Pathways, Stress Pathways and Epigenetics in the Pathogenesis of Skeletal Fluorosis. Int. J. Mol. Sci..

[174] Allen E.A., Baehrecke E.H. (2020). Autophagy in animal development. Cell Death Differ..

[175] Ravanan P., Srikumar I.F., Talwar P. (2017). Autophagy: The spotlight for cellular stress responses. Life Sci..

[176] White E. (2015). The role for autophagy in cancer. J. Clin. Investig..

[177] Levy J.M.M., Towers C.G., Thorburn A. (2017). Targeting autophagy in cancer. Nat. Rev. Cancer.

[178] Fraser J., Cabodevilla A.G., Simpson J., Gammoh N. (2017). Interplay of autophagy, receptor tyrosine kinase signalling and endocytic trafficking. Essays Biochem..

[179] Barrow-McGee R., Kishi N., Joffre C., Ménard L., Hervieu A., Bakhouche B., Noval A., Mai A., Guzmán C., Robbez-Masson L. (2016). Beta 1-integrin-c-Met cooperation reveals an inside-in survival signalling on autophagy-related endomembranes. Nat. Commun..

[180] Yamamoto K., Venida A., Yano J., Biancur D., Kakiuchi M., Gupta S., Sohn A., Mukhopadhyay S., Lin E., Parker S. (2020). Autophagy promotes immune evasion of pancreatic cancer by degrading MHC-I. Nature.

[181] Gu X., Han D., Chen W., Zhang L., Lin Q., Gao J., Fanning S., Han B. (2016). SIRT1-mediated FoxOs pathways protect against apoptosis by promoting autophagy in osteoblast-like MC3T3-E1 cells exposed to sodium fluoride. Oncotarget.

[182] Li X., Meng L., Wang F., Hu X., Yu Y. (2019). Sodium fluoride induces apoptosis and autophagy via the endoplasmic reticulum stress pathway in MC3T3-E1 osteoblastic cells. Mol. Cell. Biochem..

[183] Xu L., Deng C., Zhang Y., Zhao L., Linghu Y., Yu Y. (2021). Expression of Autophagy-Related Factors LC3A and Beclin 1 and Apoptosis-Related Factors Bcl-2 and BAX in Osteoblasts Treated With Sodium Fluoride. Front. Physiol..

[184] Wang Y., Li A., Mehmood K., Hussain R., Abbas R.Z., Javed M.T., Chang Y.-F., Hu L., Pan J., Li Y. (2021). Long-term exposure to the fluoride blocks the development of chondrocytes in the ducks: The molecular mechanism of fluoride regulating autophagy and apoptosis. Ecotoxicol. Environ. Saf..

[185] Ma L., Zhang R., Li D., Qiao T., Guo X. (2021). Fluoride regulates chondrocyte proliferation and autophagy via PI3K/AKT/mTOR signaling pathway. Chem. Interact..

[186] Suzuki M., Bartlett J.D. (2014). Sirtuin1 and autophagy protect cells from fluoride-induced cell stress. Biochim. Biophys. Acta.

[187] Lei S., Zhang Y., Zhang K.-Q., Li J. (2016). [In vivo and in vitro experimental study on the effect of fluoride-induced autophagy in rat HAT-7 cell line]. Shanghai Kou Qiang Yi Xue.

[188] Urut F., DeDe S., Yuksek V., Cetin S., Usta A., Taspinar M. (2021). In Vitro Evaluation of the Apoptotic, Autophagic, and Necrotic Molecular Pathways of Fluoride. Biol. Trace Elem. Res..

[189] Guo Q., Sun Z., Niu R., Manthari R.K., Yuan M., Yang K., Cheng M., Gong Z., Wang J. (2020). Effect of arsenic and/or fluoride gestational exposure on renal autophagy in offspring mice. Chemosphere.

[190] Tian X., Xie J., Chen X., Dong N., Feng J., Gao Y., Tian F., Zhang W., Qiu Y., Niu R. (2019). Deregulation of autophagy is involved in nephrotoxicity of arsenite and fluoride exposure during gestation to puberty in rat offspring. Arch. Toxicol..

[191] Kuang P., Deng H., Liu H., Cui H., Fang J., Zuo Z., Deng J., Li Y., Wang X., Zhao L. (2018). Sodium fluoride induces splenocyte autophagy via the mammalian targets of rapamycin (mTOR) signaling pathway in growing mice. Aging.

[192] Zhao Y., Li Y., Wang J., Manthari R.K., Wang J. (2018). Fluoride induces apoptosis and autophagy through the IL-17 signaling pathway in mice hepatocytes. Arch. Toxicol..

[193] Tang S., Zhang S., Chen W., Quan C., Duan P., Huang W., Wang A., Yang K. (2017). [Effects of fluoride on autophagy level in human neuroblastoma SH-SY5Y cells]. Wei Sheng Yan Jiu.

[194] Zhang C., Huo S., Fan Y., Gao Y., Yang Y., Sun D. (2020). Autophagy May Be Involved in Fluoride-Induced Learning Impairment in Rats. Biol. Trace Elem. Res..

[195] Coniglio S., Miller I., Symons M., Segall J.E. (2016). Coculture Assays to Study Macrophage and Microglia Stimulation of Glioblastoma Invasion. J. Vis. Exp..

[196] Prionisti I., Buhler L.H., Walker P.R., Jolivet R.B. (2019). Harnessing Microglia and Macrophages for the Treatment of Glioblastoma. Front. Pharmacol..

[197] Ye X.-Z., Xu S.-L., Xin Y.-H., Yu S.-C., Ping Y.-F., Chen L., Xiao H.-L., Wang B., Yi L., Wang Q.-L. (2012). Tumor-Associated Microglia/Macrophages Enhance the Invasion of Glioma Stem-like Cells via TGF-β1 Signaling Pathway. J. Immunol..

[198] Yeung Y.T., McDonald K.L., Grewal T., Munoz L. (2013). Interleukins in glioblastoma pathophysiology: Implications for therapy. Br. J. Pharmacol..

[199] Yuzhalin A. (2014). Interleukins in Cancer Biology: Their Heterogeneous Role.

[200] Yan L., Liu S., Wang C., Wang F., Song Y., Yan N., Xi S., Liu Z., Sun G. (2013). JNK and NADPH Oxidase Involved in Fluoride-Induced Oxidative Stress in BV-2 Microglia Cells. Mediat. Inflamm..

[201] Yang L., Jin P., Wang X., Zhou Q., Lin X., Xi S. (2018). Fluoride activates microglia, secretes inflammatory factors and influences synaptic neuron plasticity in the hippocampus of rats. Neurotoxicology.

[202] Wang J., Yue B., Zhang X., Guo X., Sun Z., Niu R. (2021). Effect of exercise on microglial activation and transcriptome of hippocampus in fluorosis mice. Sci. Total Environ..

[203] Aggarwal B.B. (2003). Signalling pathways of the TNF superfamily: A double-edged sword. Nat. Rev. Immunol..

[204] Cuny E., Loiseau H., Penchet G., Ellie E., Arsaut J., Vital A., Vincendeau P., Demotes-Mainard J. (2002). Association of elevated glial expression of interleukin-1β with improved survival in patients with glioblastomas multiforme. J. Neurosurg..

[205] Sharma V., Dixit D., Ghosh S., Sen E. (2011). COX-2 regulates the proliferation of glioma stem like cells. Neurochem. Int..

[206] Sarkar S., Yong V.W. (2009). Inflammatory cytokine modulation of matrix metalloproteinase expression and invasiveness of glioma cells in a 3-dimensional collagen matrix. J. Neuro Oncol..

[207] Yeung Y.T., Bryce N., Adams S., Braidy N., Konayagi M., McDonald K.L., Teo C., Guillemin G., Grewal T., Munoz L. (2012). p38 MAPK inhibitors attenuate pro-inflammatory cytokine production and the invasiveness of human U251 glioblastoma cells. J. Neuro Oncol..

[208] Sakuma S., Sawamura Y., Tada M., Aida T., Abe H., Suzuki K., Taniguchi N. (1993). Responses of human glioblastoma cells to human natural tumor necrosis factor-alpha: Susceptibility, mechanism of resistance and cytokine production studies. J. Neuro Oncol..

[209] Balkwill F. (2006). TNF-α in promotion and progression of cancer. Cancer Metastasis Rev..

[210] Ryu J., Ku B., Lee Y., Jeong J., Kang S., Choi J., Yang Y., Lee D., Roh G., Kim H. (2011). Resveratrol Reduces TNF-α-induced U373MG Human Glioma Cell Invasion through Regulating NF-κB Activation and uPA/uPAR Expression. Anticancer. Res..

[211] Nabors L., Suswam E., Huang Y., Yang X., Johnson M., King P. (2003). Tumor Necrosis Factor α Induces Angiogenic Factor Up-Regulation in Malignant Glioma Cells: A Role for RNA Stabilization and HuR. Cancer Res..

[212] Roomi M.W., Kalinovsky T., Rath M., Niedzwiecki A. (2017). Modulation of MMP-2 and MMP-9 secretion by cytokines, inducers and inhibitors in human glioblastoma T-98G cells. Oncol. Rep..

[213] Chopra S., Overall C.M., Dufour A. (2019). Matrix metalloproteinases in the CNS: Interferons get nervous. Cell. Mol. Life Sci..

[214] Mu N., Gu J., Liu N., Xue X., Shu Z., Zhang K., Huang T., Chu C., Zhang W., Gong L. (2018). PRL-3 is a potential glioblastoma prognostic marker and promotes glioblastoma progression by enhancing MMP7 through the ERK and JNK pathways. Theranostics.

[215] Sincevičiūtė R., Vaitkienė P., Urbanavičiūtė R., Steponaitis G., Tamašauskas A., Skiriutė D. (2018). MMP2 is associated with glioma malignancy and patient outcome. Int. J. Clin. Exp. Pathol..

[216] Zhang H., Ma Y., Wang H., Xu L., Yu Y. (2019). MMP-2 expression and correlation with pathology and MRI of glioma. Oncol. Lett..

[217] Zhou W., Yu X., Sun S., Zhang X., Yang W., Zhang J., Zhang X., Jiang Z. (2019). Increased expression of MMP-2 and MMP-9 indicates poor prognosis in glioma recurrence. Biomed. Pharmacother..

[218] Slompo C., Buzalaf C.P., Damante C.A., Martins G.M., Hannas A.R., Buzalaf M.A.R., Oliveira R.C. (2012). Fluoride modulates preosteoblasts viability and matrix metalloproteinases-2 and -9 activities. Braz. Dent. J..

[219] Wang H., Zhao W., Tan P., Liu J., Zhao J., Zhou B. (2017). The MMP-9/TIMP-1 System is Involved in Fluoride-Induced Reproductive Dysfunctions in Female Mice. Biol. Trace Elem. Res..

[220] Quadri J.A., Sarwar S., Pinky, Kar P., Singh S., Mallick S.R., Arava S., Nag T.C., Roy T.S., Shariff A. (2018). Fluoride induced tissue hypercalcemia, IL-17 mediated inflammation and apoptosis lead to cardiomyopathy: Ultrastructural and biochemical findings. Toxicology.

[221] Ichikawa T., Otani Y., Kurozumi K., Date I. (2016). Phenotypic Transition as a Survival Strategy of Glioma. Neurol. Med. Chir..

[222] Höring E., Harter P., Seznec J., Schittenhelm J., Bühring H.-J., Bhattacharyya S., von Hattingen E., Zachskorn C., Mittelbronn M., Naumann U. (2012). The “go or grow” potential of gliomas is linked to the neuropeptide processing enzyme carboxypeptidase E and mediated by metabolic stress. Acta Neuropathol..

[223] Godlewski J., Nowicki M., Bronisz A., Nuovo G., Palatini J., De Lay M., Van Brocklyn J., Ostrowski M., Chiocca E., Lawler S. (2010). MicroRNA-451 regulates LKB1/AMPK signaling and allows adaptation to metabolic stress in glioma cells. Mol. Cell.

[224] Godlewski J., Bronisz A., O Nowicki M., Chiocca E.A., Lawler S. (2010). microRNA-451: A conditional switch controlling glioma cell proliferation and migration. Cell Cycle.

[225] Bao Z., Chen K., Krepel S., Tang P., Gong W., Zhang M., Liang W., Trivett A., Zhou M., Wang J.M. (2019). High Glucose Promotes Human Glioblastoma Cell Growth by Increasing the Expression and Function of Chemoattractant and Growth Factor Receptors. Transl. Oncol..

[226] Pain G. (2018). Fluoride Causes Diabetes 2018 Update. https://www.researchgate.net/publication/328249196_Fluoride_Causes_Diabetes_2018_Update.

[227] Fluegge K. (2016). Community water fluoridation predicts increase in age-adjusted incidence and prevalence of diabetes in 22 states from 2005 and 2010. J. Water Health.

[228] Trevizol J.S., Buzalaf N.R., Dionizio A., Delgado A.Q., Cestari T.M., Bosqueiro J.R., Magalhães A.C., Buzalaf M.A.R. (2020). Effects of low-level fluoride exposure on glucose homeostasis in female NOD mice. Chemosphere.

[229] Verma R., Sherlin D.G. (2002). Sodium fluoride-induced hypoproteinemia and hypoglycemia in parental and F1-generation rats and amelioration by vitamins. Food Chem. Toxicol..

[230] Lombarte M., Fina B.L., Lupo M., Buzalaf M.A., Rigalli A. (2013). Physical exercise ameliorates the toxic effect of fluoride on the insulin–glucose system. J. Endocrinol..

[231] Hu C.-Y., Ren L.-Q., Li X.-N., Wu N., Li G.-S., Liu Q.-Y., Xu H. (2012). Effect of Fluoride on Insulin Level of Rats and Insulin Receptor Expression in the MC3T3-E1 Cells. Biol. Trace Elem. Res..

[232] Gong Y., Ma Y., Sinyuk M., Loganathan S., Thompson R.C., Sarkaria J.N., Chen W., Lathia J.D., Mobley B.C., Clark S.W. (2015). Insulin-mediated signaling promotes proliferation and survival of glioblastoma through Akt activation. Neuro Oncol..

[233] Hakuno F., Takahashi S.-I. (2018). 40 YEARS OF IGF1: IGF1 receptor signaling pathways. J. Mol. Endocrinol..

[234] Schlenska-Lange A., Knüpfer H., Lange T.J., Kiess W., Knüpfer M. (2008). Cell proliferation and migration in glioblastoma multiforme cell lines are influenced by insulin-like growth factor I in vitro. Anticancer. Res..

[235] Tirrò E., Massimino M., Romano C., Martorana F., Pennisi M.S., Stella S., Pavone G., Di Gregorio S., Puma A., Tomarchio C. (2021). Prognostic and Therapeutic Roles of the Insulin Growth Factor System in Glioblastoma. Front. Oncol..

[236] Lobo J., Leite A., Pereira H., Fernandes M., Peres-Buzalaf C., Sumida D., Rigalli A., Buzalaf M. (2015). Low-Level Fluoride Exposure Increases Insulin Sensitivity in Experimental Diabetes. J. Dent. Res..

[237] Lupo M., Buzalaf M.A.R., Rigalli A. (2010). Effect of Fluoridated Water on Plasma Insulin Levels and Glucose Homeostasis in Rats with Renal Deficiency. Biol. Trace Elem. Res..

[238] Trivedi N., Mithal A., Gupta S., Godbole M. (1993). Reversible impairment of glucose tolerance in patients with endemic fluorosis Fluoride Collaborative Study Group. Diabetologia.

[239] Turner C.H., Garetto L.P., Dunipace A.J., Zhang W., Wilson M.E., Grynpas M.D., Chachra D., McClintock R., Peacock M., Stookey G.K. (1997). Fluoride Treatment Increased Serum IGF-1, Bone Turnover, and Bone Mass, but Not Bone Strength, in Rabbits. Calcif. Tissue Int..

[240] Gutowska I., Baranowska-Bosiacka I., Goschorska M., Kolasa A., Łukomska A., Jakubczyk K., Dec K., Chlubek D. (2015). Fluoride as a factor initiating and potentiating inflammation in THP1 differentiated monocytes/macrophages. Toxicol. Vitr..

[241] Fournier T., Riches D., Winston B., Rose D., Young S., Noble P., Lake F., Henson P. (1995). Divergence in macrophage insulin-like growth factor-I (IGF-I) synthesis induced by TNF-alpha and prostaglandin E2. J. Immunol..

[242] Chao M., Liu N., Sun Z., Jiang Y., Jiang T., Xv M., Jia L., Tu Y., Wang L. (2021). TGF-β Signaling Promotes Glioma Progression Through Stabilizing Sox9. Front. Immunol..

[243] Rodón L., Gonzàlez-Juncà A., Inda M.d.M., Sala-Hojman A., Martínez-Sáez E., Seoane J. (2014). Active CREB1 promotes a malignant TGFβ2 autocrine loop in glioblastoma. Cancer Discov..

[244] Liu X.-L., Song J., Liu K.-J., Wang W.-P., Xu C., Zhang Y.-Z., Liu Y. (2015). Role of inhibition of osteogenesis function by Sema4D/Plexin-B1 signaling pathway in skeletal fluorosis in vitro. J. Huazhong Univ. Sci. Technol..

[245] Yang C., Wang Y., Xu H. (2017). Fluoride Regulate Osteoblastic Transforming Growth Factor-β1 Signaling by Mediating Recycling of the Type I Receptor ALK5. PLoS ONE.

[246] Zhao Y., Li Y., Gao Y., Yuan M., Manthari R.K., Wang J., Wang J. (2018). TGF-β1 acts as mediator in fluoride-induced autophagy in the mouse osteoblast cells. Food Chem. Toxicol..

[247] Ouyang T., Qin Y., Luo K., Han X., Yu C., Zhang A., Pan X. (2021). miR-486-3p regulates CyclinD1 and promotes fluoride-induced osteoblast proliferation and activation. Environ. Toxicol..

[248] Lütfioğlu M., Sakallıoğlu E., Sakallıoğlu U., Gülbahar M., Muğlalı M., Baş B., Aksoy A. (2012). Excessıve fluorıde ıntake alters the MMP-2, TIMP-1 and TGF-β levels of perıodontal soft tıssues: An experımental study ın rabbits. Clin. Oral Investig..

[249] Li Y., Zhao Y., Wang J., Cheng M., Wang J. (2020). Interleukin 17A deficiency alleviates fluoride-induced testicular injury by inhibiting the immune response and apoptosis. Chemosphere.

[250] Wang Y., Yuan R., Sun Y.-P., Lee T.-J., Shah G. (2003). Antiproliferative action of calcitonin on lactotrophs of the rat anterior pituitary gland: Evidence for the involvement of transforming growth factor beta 1 in calcitonin action. Endocrinology.

[251] Chen S., Li B., Lin S., Huang Y., Zhao X., Zhang M., Xia Y., Fang X., Wang J., Hwang S.-A. (2013). Change of urinary fluoride and bone metabolism indicators in the endemic fluorosis areas of southern china after supplying low fluoride public water. BMC Public Health.

[252] Shashi A., Singla S. (2013). Parathyroid function in osteofluorosis. World J. Med. Sci..

[253] Zhang X., Zhang Y., Xi S., Cheng G., Guo X. (2012). [The effect of different fluoride concentrations on the expression of transforming growth factor-beta1 in ameloblast of rat incisor]. Hua Xi Kou Qiang Yi Xue Za Zhi.

[254] Suzuki M., Shin M., Simmer J., Bartlett J. (2014). Fluoride Affects Enamel Protein Content *via* TGF-β1-mediated KLK4 Inhibition. J. Dent. Res..

[255] Luo P.-P., Xu H.-S. (2018). Effect of overdose fluoride on the expression of TGF-β3 in rat incisor. Shanghai Kou Qiang Yi Xue.

[256] Nauman P. (2015). Thyroid hormones in the central nervous system (CNS) and their effect on neoplasm formation, particularly on the development and course of glioblastoma multiforme—Research hypothesis. Endokrynol. Pol..

[257] Davis P.J., Lin H.-Y., Thangirala S., Yalcin M., Tang H.-Y., Hercbergs A., Leith J.T., Luidens M.K., Ashur-Fabian O., Incerpi S. (2014). Nanotetrac targets integrin αvβ3 on tumor cells to disorder cell defense pathways and block angiogenesis. Onco Targets Ther..

[258] Mori Y., Tomonaga D., Kalashnikova A., Furuya F., Akimoto N., Ifuku M., Okuno Y., Beppu K., Fujita K., Katafuchi T. (2015). Effects of 3,3’,5-triiodothyronine on microglial functions. Glia.

[259] Hadler-Olsen E., Winberg J.-O., Uhlin-Hansen L. (2013). Matrix metalloproteinases in cancer: Their value as diagnostic and prognostic markers and therapeutic targets. Tumor Biol..

[260] Moeller L.C., Dumitrescu A.M., Refetoff S. (2005). Cytosolic Action of Thyroid Hormone Leads to Induction of Hypoxia-Inducible Factor-1α and Glycolytic Genes. Mol. Endocrinol..

[261] Ashur-Fabian O., Blumenthal D., Bakon M., Nass D., Davis P., Hercbergs A. (2013). Long-term response in high-grade optic glioma treated with medically induced hypothyroidism and carboplatin: A case report and review of the literature. Anticancer Drugs.

[262] Berghoff A.S., Wippel C., Starzer A.M., Ballarini N., Wolpert F., Bergen E., Wolf P., Steindl A., Widhalm G., Gatterbauer B. (2020). Hypothyroidism correlates with favourable survival prognosis in patients with brain metastatic cancer. Eur. J. Cancer.

[263] Schiera G., Di Liegro C., Di Liegro I. (2021). Involvement of Thyroid Hormones in Brain Development and Cancer. Cancers.

[264] Kheradpisheh Z., Mirzaei M., Mahvi A.H., Mokhtari M., Azizi R., Fallahzadeh H., Ehrampoush M.H. (2018). Impact of Drinking Water Fluoride on Human Thyroid Hormones: A Case- Control Study. Sci. Rep..

[265] Singh N., Verma K.G., Verma P., Sidhu G.K., Sachdeva S. (2014). A comparative study of fluoride ingestion levels, serum thyroid hormone & TSH level derangements, dental fluorosis status among school children from endemic and non-endemic fluorosis areas. Springerplus.

[266] Zhang S., Zhang X., Liu H., Qu W., Guan Z., Zeng Q., Jiang C., Gao H., Zhang C., Lei R. (2015). Modifying Effect of COMT Gene Polymorphism and a Predictive Role for Proteomics Analysis in Children’s Intelligence in Endemic Fluorosis Area in Tianjin, China. Toxicol. Sci..

[267] Uller R.P., Van Herle A.J., Chopra I.J. (1973). Comparison of Alterations in Circulating Thyroglobulin, Triiodothyronine and Thyroxine in Response to Exogenous (Bovine) and Endogenous (Human) Thyrotropin. J. Clin. Endocrinol. Metab..

[268] Cuddapah V.A., Robel S., Watkins S., Sontheimer H. (2014). A neurocentric perspective on glioma invasion. Nat. Rev. Neurosci..

[269] de Groot J., Sontheimer H. (2011). Glutamate and the Biology of Gliomas. Glia.

[270] Piao Y., Lu L., de Groot J. (2009). AMPA receptors promote perivascular glioma invasion via beta1 integrin-dependent adhesion to the extracellular matrix. Neuro Oncol..

[271] Colman H., Zhang L., Sulman E.P., McDonald J.M., Shooshtari N.L., Rivera A., Popoff S., Nutt C.L., Louis D.N., Cairncross J.G. (2010). A multigene predictor of outcome in glioblastoma. Neuro Oncol..

[272] Wang X.-Y., Li Y.-L., Wang H.-Y., Zhu M., Guo D., Wang G.-L., Gao Y.-T., Yang Z., Li T., Yang C.-Y. (2017). Propofol inhibits invasion and proliferation of C6 glioma cells by regulating the Ca^2+^ permeable AMPA receptor-system x_c_^−^ pathway. Toxicol. In Vitro.

[273] Yang C., Xia Z., Li T., Chen Y., Zhao M., Sun Y., Ma J., Wu Y., Wang X., Wang P. (2020). Antioxidant Effect of Propofol in Gliomas and Its Association With Divalent Metal Transporter 1. Front. Oncol..

[274] Beretta F., Bassani S., Binda E., Verpelli C., Bello L., Galli R., Passafaro M. (2009). The GluR2 subunit inhibits proliferation by inactivating Src-MAPK signalling and induces apoptosis by means of caspase 3/6-dependent activation in glioma cells. Eur. J. Neurosci..

[275] Salmaggi A., Corno C., Maschio M., Donzelli S., D’Urso A., Perego P., Ciusani E. (2021). Synergistic Effect of Perampanel and Temozolomide in Human Glioma Cell Lines. J. Pers. Med..

